# Behaviour and landscape contexts determine the effects of artificial light on two crepuscular bird species

**DOI:** 10.1007/s10980-024-01875-3

**Published:** 2024-03-26

**Authors:** Carrie Ann Adams, Colleen Cassady St. Clair, Elly C. Knight, Erin M. Bayne

**Affiliations:** 1 Department of Biological Sciences, University of Alberta, CW 405, Biological Sciences Building, Edmonton, AB Canada; 2 Department of Fish, Wildlife and Conservation Biology, Colorado State University, 1474 Campus Delivery, Fort Collins, CO USA; 3 Alberta Biodiversity Monitoring Institute, 1-107 Centennial Centre for Interdisciplinary Studies (CCIS), University of Alberta, Edmonton, AB Canada

**Keywords:** Light pollution, Artificial light at night, Avian ecology, Caprimulgiformes, Insectivores

## Abstract

**Context:**

Artificial light at night (ALAN) is increasing worldwide, with many ecological effects. Aerial insectivores may benefit from foraging on insects congregating at light sources. However, ALAN could negatively impact them by increasing nest visibility and predation risk, especially for ground-nesting species like nightjars (*Caprimulgidae*).

**Objectives:**

We tested predictions based on these two alternative hypotheses, potential foraging benefits vs potential predation costs of ALAN, for two nightjar species in British Columbia: Common Nighthawks (*Chordeiles minor*) and Common Poorwills (*Phalaenoptilus nuttallii*).

**Methods:**

We modeled the relationship between ALAN and relative abundance using count data from the Canadian Nightjar Survey. We distinguished territorial from extra-territorial Common Nighthawks based on their wingboom behaviour.

**Results:**

We found limited support for the foraging benefit hypothesis: there was an increase in relative abundance of extra-territorial Common Nighthawks in areas with higher ALAN but only in areas with little to no urban land cover. Common Nighthawks’ association with ALAN became negative in areas with 18% or more urban land cover. We found support for the nest predation hypothesis: the were strong negative associations with ALAN for both Common Poorwills and territorial Common Nighthawks.

**Conclusions:**

The positive effects of ALAN on foraging nightjars may be limited to species that can forage outside their nesting territory and to non-urban areas, while the negative effects of ALAN on nesting nightjars may persist across species and landscape contexts. Reducing light pollution in breeding habitat may be important for nightjars and other bird species that nest on the ground.

**Supplementary Information:**

The online version contains supplementary material available at 10.1007/s10980-024-01875-3.

## Introduction

Artificial light at night (ALAN) is increasing worldwide (Falchi et al. 2016; Kyba et al. 2017; Sánchez de Miguel et al. 2021; Cox et al. 2022), as are studies on its biological impacts (Rodrigo-Comino et al. 2021; Adams et al. 2021). While ALAN is typically brightest in urban environments, rural areas are becoming increasingly illuminated as more light sources are installed and more skyglow from distant sources of ALAN reflects off particles in the atmosphere back towards the earth (Min and Gaba 2014; Gaston et al. 2015; Falchi et al. 2016). Most species evolved under predictable solar and lunar cycles, which ALAN substantially alters (Gaston et al. 2014). These alterations affect biological systems from the levels of molecules to ecosystems. At the molecular level, ALAN affects gene expression (Chen et al. 2021) and hormone production (Injaian et al. 2021). A growing body of work links ALAN to changes in behaviour, such as vocalizing (Da Silva et al. 2015), sleeping (Aulsebrook et al. 2020), and foraging (Santos et al. 2010), which can combine to alter species abundance and distribution (La Sorte et al. 2017; McLaren et al. 2018; Simons et al. 2021). ALAN also impacts predator–prey relationships (Underwood et al. 2017; Ditmer et al. 2020; Nuñez et al. 2021), inter-species competition (Valeria et al. 2021), and ecosystem services, such as pollination (Knop et al. 2017; Straka et al. 2021) and seed dispersal (Lewanzik and Voigt 2014). Nocturnal and crepuscular species are thought to be more vulnerable to the negative effects of ALAN than diurnal species (Sanders et al. 2020; Ditmer et al. 2021) because they are exposed to more artificial light than diurnal species when lights turn on after sunset and artificial lights become brighter relative to ambient illumination. While the effects of ALAN are often negative, the costs and benefits can depend on the species under study (Sanders et al. 2020), geographic or landscape features (Barré et al. 2021; Camacho et al. 2021), and the spatial scale at which ALAN is measured (McLaren et al. 2018).

ALAN may provide foraging opportunities for insectivorous birds and bats by aggregating their insect prey under lights (Shields and Bildstein 1979; Bharos 1992; Foley and Wszola 2017). This type of foraging behavior has been documented around the world, but is undoubtedly subject to observation bias because birds foraging away from lights are less likely to be seen by humans (Buij and Gschweng 2017). Evidence from studies on bats suggests they can benefit by foraging on insects aggregating at lights, although not all light-attracted bat species consistently increase their activity near artificial lights and the effects of ALAN on foraging behaviour can depend on landscape context (Mathews et al. 2015). Furthermore, the long-term effects of ALAN on insect abundance have not been adequately studied (Kalinkat et al. 2021), and the benefits for aerial insectivores may diminish over time if mortality and disrupted reproduction at artificial light depletes local insect populations (Eisenbeis 2006; van Grunsven et al. 2019). Finally, the cumulative effects of many light sources over large spatial extents are relatively unknown, but ALAN may reduce insect populations over large extents by creating population sinks (van Grunsven et al. 2020), limiting dispersal (Degen et al. 2016), and creating widespread skyglow that impacts their physiology and behaviour (reviewed by Owens and Lewis 2018 and Owens et al. 2019). Therefore, landscapes with more light pollution may support fewer aerial insectivores, opposite to the prediction based on insect aggregations associated with ALAN (Eisenbeis 2006; Carannante et al. 2021).

In the context of nesting, ALAN may harm aerial insectivores by increasing the visibility of their nests, especially for ground-nesting species. Most previous studies on breeding birds and ALAN have focused on species that nest in cavities, on buildings, or in trees and generally have found no correlation between breeding densities and ALAN (Jong et al. 2015; Russ et al. 2017; Wang et al. 2021). However, cliff-nesting seabirds experienced higher predation in areas of a breeding colony exposed to artificial light (Oro et al. 2005) while a ground-nesting shorebird selected nest sites farther from artificial lights (de Molenaar et al. 2006). Ground-nesting species may be more likely to be depredated by mammals (Roos et al. 2018), and predation often increases with ALAN (Sanders et al. 2020), suggesting that changes in predator behavior may influence how ALAN affects birds. Nightjars in particular rely heavily on camouflage to avoid nest predation (Troscianko et al. 2016), and artificial light, especially broad-spectrum light produced by LEDs, has the potential to increase the visibility of camouflaged prey species (McMahon et al. 2022). Increased illumination (from moonlight or ALAN) has been shown to increase detection rates by visually orienting predators (Clarke 1983; Santos et al. 2010). Thus, increases in perceived and/or actual predation risk may cause ground-nesters to select nest sites further away from artificial lights and/or experience nest failure near ALAN.

Nightjars of the *Caprimuligidae* family may experience both the foraging benefits and predation risks of ALAN because they are crepuscular and nocturnal birds that hunt flying insects and nest on the ground. The family includes 89 species found on every continent other than Antarctica (Winkler et al. 2020). Nightjars sometimes forage under artificial lights (Shields and Bildstein 1979; Ingels et al. 1999; Jackson 2003; Foley and Wszola 2017) and species accounts suggest that this behaviour is common (Winkler et al. 2020; Woods et al. 2020; Brigham et al. 2020). However, studies of how artificial light affects their habitat use have mixed results and are confounded by urbanization. Common Nighthawk (*Chordeiles minor*) occurrence in Wisconsin was positively correlated with streetlights during the breeding season, but showed a stronger correlation with gravel rooftops, which also occur in urban areas and provide an important nesting substrate for nighthawks (Newberry 2018; Viel et al. 2020). The European Nightjar (*Caprimulgus europaeus*) and Eastern Whip-poor-will (*Antrostomus vociferus*) showed negative responses to urbanization and the associated light pollution during migratory and breeding periods (Sierro and Erhardt 2019; Korpach et al. 2022). Understanding whether foraging under artificial light occurs only in isolated cases or is common enough to influence their occurrence or abundance is important for understanding whether ALAN alters predator–prey relationships between nightjars and insects. Nightjar species that forage away from their nest sites may respond differently to ALAN for territorial behaviors, related to nesting, compared with extra-territorial behaviours, which include foraging. Species that forage and nest within the same area must balance the foraging costs and predation benefits when selecting a territory.

We evaluated the effects of ALAN on the relative abundance of two nightjar species, Common Nighthawks and Common Poorwills (*Phalaenoptilus nuttallii*), at sites surveyed in British Columbia during the Canadian Nightjar Survey. Breeding Bird Survey trends show both species’ populations declining across much of their range (Sauer et al. 2020). Both have been observed foraging under artificial lights at night (Preston 2015; Foley and Wszola 2017), suggesting a potential benefit of ALAN. Common Nighthawks defend a small nest site with a behaviour called wingbooming (Knight et al. 2021a), but vocalize frequently as they travel up to tens of kilometers to forage, allowing us to separately evaluate how ALAN affects the relative abundance of territorial and extra-territorial individuals for this species. Common Poorwills conduct all of their nesting and foraging activities within a relatively small territory (Csada and Brigham 1994)*,* with breeding individuals typically foraging within hundreds of metres from the nest site.

We weighed evidence for two hypotheses by measuring the effects of ALAN on the relative abundance of three types of nightjars (territorial Common Nighthawks, extra-territorial Common Nighthawks, and territorial Common Poorwills) over multiple spatial scales (Table 1). The hypothesis that ALAN provides a foraging benefit for nightjars would be supported by an increase in the relative abundance of extra-territorial Common Nighthawks and of territorial Common Poorwills in areas with ALAN, measured at a local scale. The hypothesis that ALAN increases nest predation risk for ground-nesting species would be supported by a decrease in the relative abundance of territorial Common Nighthawks and Common Poorwills at sites with higher ALAN, also at a local scale. Nighthawks may benefit from nesting in a dark area within a landscape where they can travel to forage under a light source. The relative abundance of territorial Common Nighthawks could support both the foraging benefit and nest predation risk hypotheses if it were negatively correlated to ALAN at the local scale, but positively correlated with ALAN at the landscape scale.

**Table 1:** Predictions associated with the foraging benefit and predation risk hypotheses

	Foraging benefit hypothesis: Artificial light at night (ALAN) provides foraging opportunities for crepuscular aerial insectivores	Nest predation cost hypothesis: ALAN light increases predation risk for crepuscular, ground-nesting species
Extra-territorial Common Nighthawks	Increased relative abundance at sites with higher *local-scale ALAN* if they forage under lights	NA
Territorial Common Nighthawks	Increased relative abundance at sites with higher *landscape-scale ALAN* if they travel to forage under artificial lights away from nest site	Decreased relative abundance at sites with higher *local-scale ALAN* if ALAN increases predation risk at the nest site
Common Poorwill	Increased relative abundance at sites with higher *local-scale ALAN* if they forage under lights	Decreased relative abundance at sites with higher *local-scale ALAN* if ALAN increases predation risk for nesting and/or foraging poorwills

## Methods

### Study area

Our study area spanned several ecoprovinces in the province of British Columbia, Canada (Demarchi 2011). The Coast and Mountains ecoprovince on the west coast has heavy rain and lush vegetation. The drier, low elevation Georgia Depression includes the heavily populated cities of Vancouver and Victoria. Moving east, the Central Interior has open grasslands and rolling plateaus, while the Southern Interior has Ponderosa pine (*Pinus ponderosa*) and Douglas fir (*Pseudotsuga menziesii*) forests as well as urban areas in the Okanagan Valley. In the eastern part of our study area, the Southern Interior Mountains host high peaks and thick forests, with wetlands and rivers in the valleys. For our analysis of Common Poorwills, we only included surveys conducted in the Southern Interior ecoprovince, which encompasses the species’ range within British Columbia (Woods et al. 2020), and conducted within the boundary of the Annual Crop Inventory (ACI) (Agriculture and Agri-Food Canada 2020) where the most detailed land use/land cover data were available.

### Nightjar surveys

Community scientists conducted roadside point counts for the Canadian Nightjar Survey (CNS) every June and July from 2014 to 2020. Routes were generated using random starting points from all possible roadside locations and random survey directions. Not all routes were surveyed and volunteer preference influenced which routes were taken. Each survey route consisted of six to ten stations spaced approximately 1.6 km apart. Surveys began 30 min before sunset and consisted of 6-min observation periods at each station. Volunteers recorded each individual nightjar, the species, and the detection type (visual, wingboom, or vocalization) for each 1-min interval within each 6-min observation period. Additional information on survey protocol is available in the BC Nightjar Survey annual reports (WildResearch 2019). We downloaded CNS data from the NatureCounts web site (Birds Canada and WildResearch 2021).

Male Common Nighthawks establish a territory and defend approximately 400 m around their nest using aerial displays called wingbooms (Rust 1947; Knight et al. 2021a). During their peak activity period, civil twilight (Sidler 2017), wingboom rate is high and we assumed nighthawks heard vocalizing, but not wingbooming, were extra-territorial. We tested this assumption in our detection probability model (described in the Data Analysis section). Studies have found that habitat associations are different for wingbooming and non-wingbooming nighthawks, and they are consistent with habitat requirements for nesting and foraging, respectively (Knight and Bayne 2017; Knight et al. 2021b).

### Predictors of nightjar relative abundance

We measured all landscape predictors in three buffer sizes: 400, 1600, and 6400 m, corresponding to the buffer sizes used for another study of Common Nighthawks in Canada (Knight et al. 2022). The previous study included buffer sizes ranging from the smallest territory radius (~ 100 m) to the largest known home range radius (~ 12 km) for nighthawks in the Boreal Forest of Alberta. We only included three of the six buffer sizes used by Knight et al. (2022) to allow for model convergence, as explained below when we describe the relative abundance models. We refer to these buffer sizes as scales, which we define as the spatial extent over which we measured landscape features (McGarigal et al. 2016). We used 400 m as the smallest buffer size because the artificial light estimates, described below, have a grain size of approximately 300 × 463 m in our study area, preventing us from measuring artificial light in smaller buffer sizes. We used 6400 m as the largest buffer size because variance in ALAN measurements among surveys was substantially lower for the 12,800 m than the 6400 m buffer.

We used estimates of artificial light at night derived from the Visible Infrared Imaging Radiometer Suite Day/Night Band sensor on the Suomi Polar-orbiting Partnership Satellite (Cao et al. 2014). The sensor measures light shining upwards from a light source, light reflected off of the ground, and upward-scattered skyglow, which is theoretically similar to the downward scattered skyglow in the same location, especially for light emitted at near-horizontal angles (Sànchez de Miguel et al. 2020). We used the annual composites from the Earth Observation Group’s VIIRS Nighttime Light Products (VNL) (Elvidge et al. 2017) because they removed natural light in the aurora zone more effectively than the annual composites from NASA’s Black Marble (Román et al. 2018; Online Resource 1, Figure S4). For our analysis, we created an annual composite for each study year by calculating each pixel’s mean of Version 2 (V2) for that survey year and Version 1 (V1) for 2016. V1 is available for only 2015 and 2016, (Elvidge et al. 2017), while V2 is available for all years between 2012 and 2020 (Elvidge et al. 2021), but misses many dim light sources in our study area that were found in V1 (Online Resource 1, Figure S2). We conducted a sensitivity analysis to determine whether different versions of the annual composite (V1 2016 or V2 for the survey year) substantially changed the posterior distributions of our coefficient estimates.

We included land use and land cover types that were positively or negatively associated with Common Nighthawk or Common Poorwill habitat use in previous studies (Online Resource 1, Table S1). For Common Nighthawks, these included burned or harvested forest, water or wetlands, grassland, agriculture, and urban land cover (Ng 2009; Farrell et al. 2017; Newberry and Swanson 2018; Farrell et al. 2019; Viel et al. 2020; Knight et al. 2021b). The only study of Common Poorwill habitat associations in the northern part of their range showed positive relationships with native prairie and low-vegetation grassland or rangeland (Macdonald et al. 2003). For both species’ analyses, we used the Annual Crop Inventory (ACI) to classify proportional cover of urban, cropland, pasture, and water/wetland (Agriculture and Agri-Food Canada 2020). We classified each pixel based on its most frequent value across all study years (2014–2020). In areas that were not classified as water, wetland, cropland, pasture, or urban by ACI, we used the BC Vegetation Resource Inventory (VRI) from 2020 to measure the proportion cover of sparse forest, shrubland, and grassland (BC Ministry of Forests 2020).

We also included temporal and geographic covariates that potentially influence nightjar activity periods and occurrence or abundance. As temporal covariates, we included sun angle, sun angle squared, day of year, day of year squared, and lunar illumination, similarly to other authors (Brigham and Barclay 1992; Brigham et al. 1999; Jetz et al. 2003; Woods and Brigham 2008; Sidler 2017). All analyses were conducted in *R 4.1.1.* We measured sun angle using the R package *suncalc* (Thieurmer and Elmarhraoui 2019) and lunar illumination using the R package *moonlit* (Śmielak 2023). We did not find geographic or topographic predictors of nightjar abundance in the literature, so we evaluated the impact of elevation, slope, latitude, longitude, and their quadratic terms on the counts of each type of nightjar in GLMs before including them in our primary model. First, we used the *glm* function and the *glm.nb* function in the *MASS* package to compare a negative binomial to a Poisson GLM using all geographic predictors and selected the model form with the lowest Akaike Information Criteria (AIC) (Venables and Ripley 2002). Then, we used the *dredge* function from the *MuMln* package (Bartoń 2022) to test for effects of all geographic predictors and selected the model with the with delta AIC < 2.0, limiting the number of predictors in each candidate model to four. We included these geographic predictors in our relative abundance models. To account for nest site fidelity in the analyses of territorial Common Nighthawks and Common Poorwills, we included as a predictor the mean number of territorial individuals counted in previous surveys at the same station within the same year or during the previous year. A positive coefficient estimate for this predictor could also indicate that there are other processes causing more consistent abundance of nightjars within sites across years than predicted by the ALAN and landscape features in our model. These processes could be endogenous (e.g. natal dispersal) or exogenous (e.g. important predictors missing from our model that remain consistent within sites across years).

### Relative abundance models

Assuming equal detection probability across surveys, the number of individuals counted in each survey represents a constant, but unknown, proportion of all of the individuals present. The count in each survey thus represents abundance relative to other surveys, which we defined as relative abundance. We tested this assumption by removing surveys in which detection probability was estimated to be < 90%, as we describe below. We conducted all analyses separately for territorial (wingbooming) Common Nighthawks, extra-territorial (vocalizing but not wingbooming) Common Nighthawks, and territorial (vocalizing) Common Poorwills.

We used a multi-step Bayesian modelling process to choose the most appropriate model form, identify the most predictive scale for each landscape covariate, and then estimate the effect of each covariate. First, we used DIC comparison to identify which model form best fit our data (Online Resource 1, Table S2). Second, we used Bayesian latent indicator scale selection (BLISS) to select the buffer size at which each covariate best explained relative abundance (Stuber et al. 2017). BLISS is scale-selection procedure that evaluates all combinations of covariates and scales within a single model run, rather than using separate models to select the optimal scale for each covariate independently or to select a single optimal scale for all covariates. BLISS generated a joint posterior distribution for two coefficients for each landscape covariate: (a) the effect estimate, which represented the log of the expected change in nightjar count per unit change in the covariate; and (b) the scale of effect, which represents the buffer size at which the covariate best explained the observed nightjar counts. We identified the spatial scale of the effect of each covariate as the buffer size selected in the largest proportion of the posterior distribution. In cases where a landscape covariate had a positive effect when one scale was selected, but a negative effect when another was selected, we included both scales as separate covariates in our final model. To identify these cases, we compared the effect estimates for each covariate from samples of the joint posterior distribution from the BLISS model that selected each scale. To ensure that we identified the most explanatory spatial scale of effect for our covariates of interest, we refit the BLISS model for ALAN, urban land cover, and their interaction with all other landscape covariates measured at their selected scales. We then fit the relative abundance model with all covariates measured at their selected scale or scales to finalize the estimates for the effect of each covariate. All predictors were included in the final model. All Bayesian models were fit using JAGS (Plummer 2003) and the *R2Jags* package (Su and Yajima 2021), using three chains with 12,000 iterations each and 3,000 burn-in iterations, for a total for 27,000 samples of the joint posterior distribution. After burn-in, we retained all samples in the chains because thinning would likely reduce the precision of our parameter estimates (Link and Eaton 2012).

We used two procedures to test whether our model could correctly identify the marginal effects of highly correlated covariates. After completing the analysis for each nightjar species and behaviour, we used the coefficient values and scales of effect estimated by our models to simulate nightjar counts for each survey. We then refit the model with these simulated data to confirm that the coefficients used to simulate the data were within the 95% credible intervals (CIs) estimated by the model. We also plotted the correlation among the three correlated covariates (urban land cover, ALAN, and the interaction between the two) across samples of the posterior distribution to determine if the presence of multicollinearity masked an important effect of one of our covariates (McElreath 2019).

We initially fit the BLISS models with six buffer sizes (400, 800, 1600, 3200, 6400, and 12,800 m), but they did not converge, likely because of spatial autocorrelation across the six buffer sizes. We thinned our analysis to use three representative buffer sizes to approximate the scale of effect as local (400 m), intermediate (1600 m), or landscape (6400 m), recognizing that the specific scale at which nighthawks perceive and respond to each predictor was not precisely identified by our model and likely varies across our study area and over time.

We included two post-hoc analyses to further investigate unexpected results and test our predictions. We modeled the effects of local and landscape-scale ALAN when both were included as separate covariates in the same relative abundance model for territorial nighthawks. We removed four stations that were surveyed many times across the study period from our model for territorial nighthawks. We provide further justification and explanation of these post-hoc analyses in our results section.

To describe how ALAN influenced the relative abundance of nighthawks in our model, we calculated the mean and 95% credible intervals (CIs) of the posterior predictions for the expected nightjar count as ALAN value increased from 0 to the 95th percentile value recorded in our dataset at the selected scale. We described the relationship between ALAN and relative abundance at varying proportions of urban land cover, including median, mean, and high (95th percentile) of urban land cover within the selected buffer size. To avoid interpreting model outputs beyond the range of ALAN values that exist in our data at each urban land cover level, we limited these descriptions to the 99th percentile ALAN values that occurred at surveys with urban land cover equal to or less than the urban land cover proportion being referenced. When we calculated the expected number of nighthawks, we set all other covariates to their mean values, unless otherwise specified.

### Detection probability models

We modeled individual detection rate to determine whether the influence of ALAN on detection probability could bias our estimate of ALAN on relative abundance. Using the minute-by-minute detection data for each individual nightjar, we modeled the effect of artificial light and temporal covariates on the number of minutes (out of six) in which each individual was detected using a binomial GLM. We used this model to predict the probability that an individual, if present, would be detected in each survey. We modeled this detection probability separately for extra-territorial Common Nighthawks, territorial Common Nighthawks, and Common Poorwills. In a sensitivity analysis, we removed surveys with < 90% detection probability and refit the relative abundance models. We compared the resulting coefficient estimates for ALAN, urban, and their interaction with those from the full model to determine if they influenced the scale or direction of the estimated effects.

## Results

### Survey results

We included 6577 surveys conducted at 1806 unique survey stations in British Columbia between 2014 and 2020. Volunteers recorded wingbooming Common Nighthawks in 973 of these surveys (15%) and non-wingbooming Common Nighthawks in 1,569 surveys (24%). In surveys where wingbooming nighthawk were observed, their mean count was 1.71 (SD = 1.16). In surveys with non-wingbooming nighthawks, their mean count was 1.67 (SD = 1.11). Common Poorwills were recorded during 236 (8%) of the 2,737 surveys within the Southern Interior ecoprovince, with a mean count of 1.5 (SD = 0.79) individuals in surveys where they were observed. Common Poorwills were also observed in 11 surveys outside of their traditional species range, in the south-eastern corner of the province along the Kootenay River in the Rocky Mountains. We did not include these 11 surveys in our relative abundance model for Common Poorwills.

### ALAN and urban land cover estimates

**Fig. 1:**
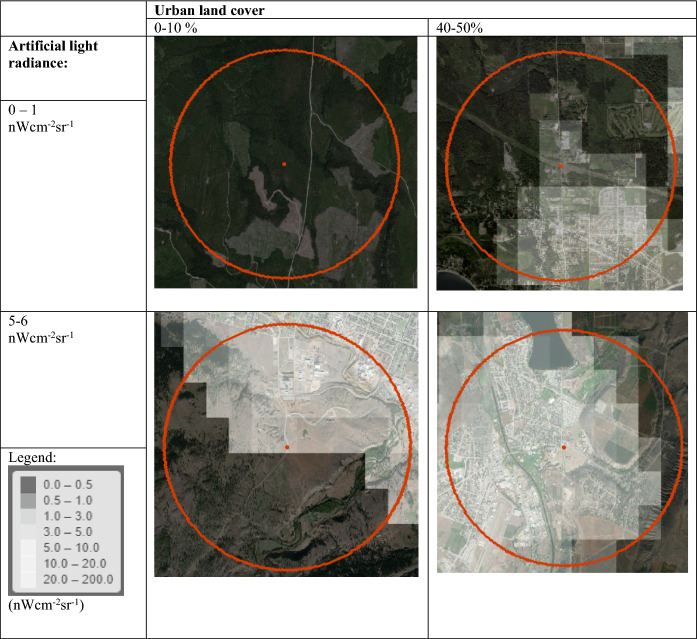
Examples of artificial light radiance values and urban land cover in a 1600-m buffer. We calculated mean radiance within a 1600-m buffer using the average of the Earth Observations Groups V1 annual composite for 2016 and V2 annual composite for the survey year. The red points represent survey points and red circles represent a 1600-m radius. Pixels within these buffers with artificial light but no anthropogenic structures likely show skyglow, the reflection of the light off of particles in the atmosphere

Artificial light estimates were low in most surveys, with median values of 0, 0.04, and 0.17 nWcm^−2^sr^−1^ for the 400, 1600, and 6400-m buffer sizes, respectively. The 95th percentile values were 6.18, 6.30, and 6.13 nWcm^−2^sr^−1^. For reference, 1600-m buffers with less than 1 nWcm^−2^sr^−1^ showed an isolated light source or overlapped a small, dimly lit settlement (Fig. 1). A 1600-m buffer with 6 nWcm^−2^sr^−1^ typically included a small settlement or the edge of a town. Median percent urban land cover was 7.23%, 3.75%, and 3.37% for the three buffer sizes, and the 95th percentile values were 56%, 42%, and 35%. Buffers of 1600 m that had 40–50% urban land cover typically overlapped a small settlement or town.

ALAN and proportion of urban land cover had Pearson’s correlation coefficients of 0.56, 0.63, and 0.85 for the 400, 1600, and 6400-m buffer sizes, respectively, across all surveys. For the subset of surveys in the Common Poorwill range, the correlation coefficients were 0.68, 0.78, and 0.86, respectively. Our model identified the correct spatial scales and coefficient values within the 95% CIs when we fit it using relative abundance values simulated from our coefficient estimates, suggesting that our model adequately estimated the marginal effects of ALAN and urban land cover despite their correlation (Online Resource 1, Figure S3).

ALAN estimates likely included direct illumination and skyglow. Where positive ALAN values occurred in pixels near urban land cover but with no plausible light sources, ALAN estimates may have included light scattered through the atmosphere and upwards towards the satellite. This upward scatter theoretically and empirically correlates with skyglow, artificial light scattered towards the ground, within a pixel of the VIIRS night-time light products (Sanchez de Miguel et al. 2020).

### Relative abundance models

In the preliminary modeling stages, we identified elevation, elevation squared, slope squared, and latitude squared as the combination of predictors with the lowest AIC in the preliminary count model for extra-territorial Common Nighthawks. The lowest AIC model for territorial Common Nighthawks was similar, but included the linear term for latitude instead of the quadratic term. For Common Poorwills, the lowest AIC model included longitude, longitude squared, slope, and slope squared. We included these covariates in their respective relative abundance models. Among the candidate model forms for the Bayesian relative abundance model (zero-inflated Poisson, negative binomial, and Poisson), we selected the negative binomial because it had the lowest DIC for all three nightjar groups (Online Resource 1, Table S2). For all three analyses, our results were insensitive to the version of the EOG annual composite (V1, V2, or the mean of V1 and V2) used to measure ALAN (Online Resource 1, Figure S4).

### Extra-territorial Common Nighthawks

**Fig. 2:**
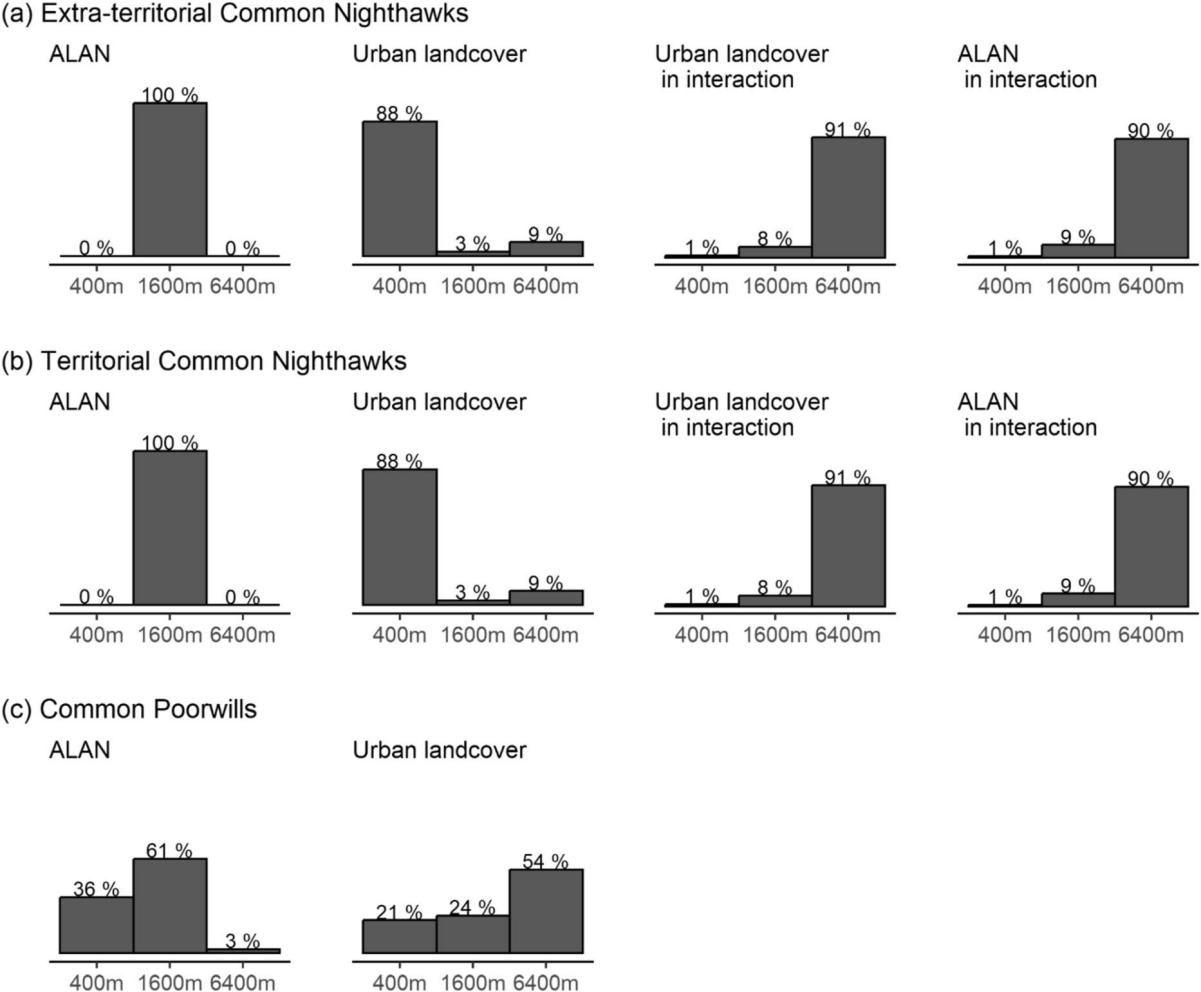
Proportions of the posterior selecting each spatial scale for ALAN, urban, and their interactions in the BLISS models. The BLISS model generates a posterior distribution for each covariate for the buffer size that best explains the relative abundance of nightjars. We tested three buffer sizes: 400, 1600, and 6400 m. Bars show the percent of the 27,000 samples of the posterior distribution that selected each buffer size

The BLISS models revealed that the relative abundance of extra-territorial Common Nighthawks was best explained by ALAN measured at the landscape scale (6400 m), but with an interaction with land cover. This landscape scale was selected both for the main effect of ALAN (96% of the posterior) and for ALAN in interaction with urban land cover (98% of the posterior) (Fig. 2a; Online Resource 1, Figure S5 and Table S3). The BLISS model also selected the landscape scale for the main effect of urban land cover, and the intermediate scale (1600 m) for its interaction with ALAN.

**Fig. 3:**
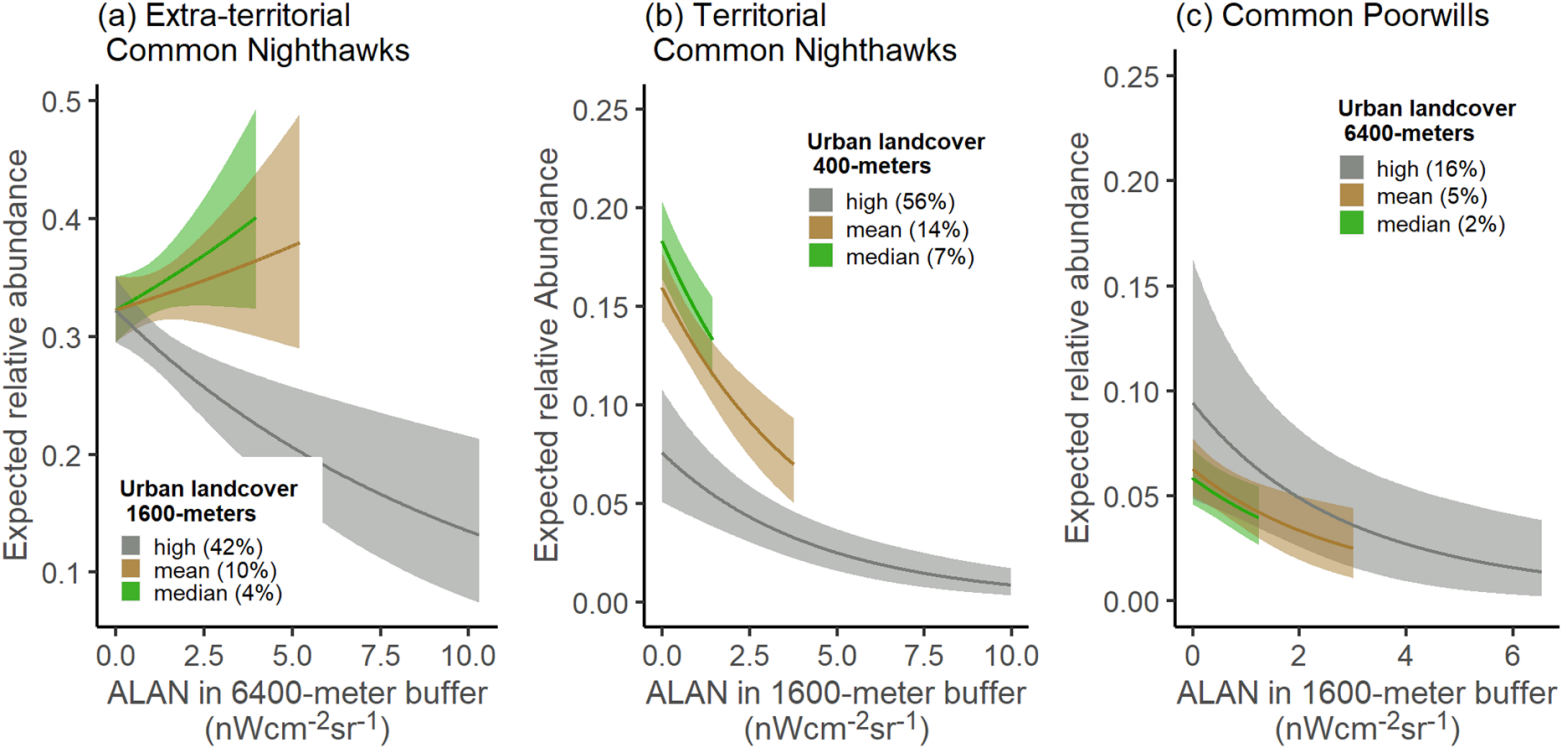
Model predications of relative abundance across ALAN levels at varying levels of urban land cover. Results for (a) Extra-territorial Common Nighthawks, (b) Territorial Common Nighthawks, and (c) Common Poorwills. The high value of urban land cover shown is the 95th percentile within the selected buffer sizes. For each proportion urban land cover shown, we included model outputs up to the 99th percentile of ALAN values in surveys with up to and including that proportion of urban land cover. We set all other covariates to their mean values, unless otherwise specified

The relationship between the relative abundance of extra-territorial Common Nighthawks and ALAN switched from positive to negative when urban land cover at the intermediate scale exceeded 18% (95% CI 3%, 30%) (Fig. 3a). Percent of urban land cover at the intermediate scale in our survey sites had a median value of 4%, a mean of 10%, and a 95th percentile value of 42%. The 99th percentile ALAN values occurring at surveys with urban land cover equal to or less than these values were 3.98 nWcm^−2^sr^−1^ (for median urban land cover), 5.2 nWcm^−2^sr^−1^ (for mean urban land cover, and 10.3 nWcm^−2^sr^−1^ (for high urban land cover). For a survey with median urban land cover, the expected number of extra-territorial Common Nighthawks increased by 25% (− 4%, + 61%) when ALAN values increased from 0 to 3.98 nWcm^−2^sr^−1^, and the 95% CI included zero (Fig. 3a; Online Resource 1, Table S4). An 18% (− 14%, + 61%) increase in the expected relative abundance occurred where ALAN increased to 5.2 nWcm^−2^sr^−1^ in areas with mean urban land cover, where the 95% CI also overlapped zero. At the 95th percentile urban land cover, an increase to 10.3 nWcm^−2^sr^−1^ corresponded to a 59% (− 30%, − 78%) decrease in the number of extra-territorial nighthawks. The 95% CI for the main effect of urban land cover alone overlapped zero (Online Resource 1, Figure S6). In samples from the joint posterior distribution, the coefficient estimate for ALAN covaried with the coefficient estimates for urban land cover and with the interaction term between ALAN and urban land cover (Figure S8). In all samples of the posterior, the coefficient for the interaction term was negative, and either the coefficient for urban land cover or ALAN, or both, were positive. To assess the effects of these correlations, we tested our model on simulated data, using fitted coefficient estimates to simulate counts. We found that it could identify the simulated positive effect of ALAN, but could not identify the simulated positive effect of urban land cover (the 95% credible interval included the simulated value but overlapped zero).

### Territorial Common Nighthawks

The BLISS procedure selected the intermediate spatial scale for the main effect of ALAN and the landscape scale for its interaction with urban land cover (Fig. 2b). In the final model, the 95% CI for this interaction overlapped zero (Online Resource 1, Figure S6). Two scales were selected for urban land cover, with a negative effect at the local scale and a positive effect at the landscape scale in both the BLISS model and the final model (Online Resource 1, Figures S5 and S6). In the final model the 95% CI for the landscape scale overlapped zero when we removed surveys conducted in one highly sampled region, described below.

The relative abundance of territorial Common Nighthawks was negatively associated with ALAN. In areas with median urban land cover (7%) at the local scale, an increase in ALAN from 0 to 1.44 nWcm^−2^sr^−1^ corresponded to a decline in the expected number of territorial Common Nighthawks of 27% (− 36%, − 18%) (Fig. 3b; Online Resource 1, Table S4). Where urban land cover was at its mean (14%), the expected number of territorial Common Nighthawks decreased by 56% (− 69%, − 40%) when ALAN increased to 3.76 nWcm^−2^sr^−1^. In areas with high urban land cover (56%), the 99th percentile ALAN value reached 9.98 nWcm^−2^sr^−1^, and at this ALAN value the expected number of territorial nighthawks was 88% lower (− 96%, − 74%) than in surveys with high urban land cover but no detectable ALAN. The 95% CI for the interaction term between ALAN and urban land cover overlapped zero (Online Resource 1, Figure S6, Table S4).

Urban land cover was selected at both the local scale (with a negative coefficient) and the landscape scale (with a positive coefficient), but the positive effect at the landscape scale arose from the high relative abundance of territorial nighthawks in one small, highly sampled area. Fifty-six surveys at four stations, clustered < 2 km of each other on the outskirts of Victoria, accounted for 70% of the surveys where landscape-scale urban land cover was > 30%. After removing these surveys, the coefficient for landscape-scale urban land cover decreased substantially and the 95% CI overlapped zero. No other model coefficients changed substantially after removing these stations (Online Resource 1, Figure S7), except for the interaction between ALAN and urban land cover. When the stations near Victoria were removed, this interaction was stronger, but predicted relative abundance of territorial Common Nighthawks as ALAN increased at each urban landcover level changed by ≤ 11% (Table S4), compared to the predictions from the model without those stations.

We modified our relative abundance model in two ways to determine if ALAN at the landscape scale could have a positive marginal effect, after accounting for the negative effect at the intermediate scale. When we included both scales as separate covariates in our model, both had negative coefficient estimates (Online Resource 1, Table S5). When we fit a version of the model only including surveys at stations with no artificial light within 1600 m, the coefficient estimate for ALAN at the landscape scale was slightly positive, but with a very wide 95% CI that overlapped zero (Online Resource 1, Table S5).

### Common Poorwills

In the BLISS model, the spatial scale of ALAN that best explained the relative abundance of Common Poorwills was the intermediate scale (Fig. 2c). We removed the interaction term between ALAN and urban land cover in our final model for Common Poorwills because the coefficient for urban land cover was highly correlated with the interaction term, resulting in very wide 95% CIs for the coefficients for urban land cover and the interaction term (Online Resource 1, Figure S8). This correlation and the wide confidence intervals likely indicate that the model could not identify the effect or urban land cover separately from its interaction with ALAN (McElreath 2019), so we were not able to test for the presence of this interaction. To better estimate the main effect of urban land cover, we removed the interaction term from the model and repeated the BLISS procedure, which selected the intermediate scale for ALAN and the landscape scale for urban land cover (Online Resource 1, Figure S5). Removing the interaction term did not substantially change the coefficient estimate for ALAN, which was -0.30 with the interaction term included and -0.32 without it.

Relative abundance of Common Poorwills was negatively associated with ALAN. For surveys with median urban land cover (2%), the expected number of Common Poorwills declined by 32% (− 52%, − 12%) as ALAN increased from 0 nWcm^−2^sr^−1^ to 1.23 nWcm^−2^sr^−1^, its 99th percentile value at this urban land cover level (Fig. 3c; Online Resource 1, Table S4). Where urban land cover was at its mean (5%), the expected number of Common Poorwills declined by 59% (− 83%, − 26%) when ALAN increased to 3.00 nWcm^−2^sr^−1^. Where urban land cover was high (16%), ALAN reached up to 6.55 nWcm^−2^sr^−1^, which corresponded to an 84% (− 98%, − 26%) decrease in the expected number of Common Poorwills. The 95% CI of the coefficient for urban land cover overlapped zero (Online Resource 1, Figure S6, Table S4).

### Detection probability models

We found some evidence that detection probability for individual territorial Common Nighthawks was lower in sites with light pollution, but it did not influence the outcomes of our relative abundance analyses. We modeled the effects of ALAN on the vocalization rate for Common Poorwills, wingboom rate for territorial Common Nighthawks, and vocalization rate for extra-territorial Common Nighthawks. The coefficients for ALAN were negative for Common Poorwills and territorial Common Nighthawks, and the 95% CI overlapped zero for Common Poorwills but not for territorial Common Nighthawks. However, the probability that an individual, if present, would be detected within a 6-min survey (i.e. detection probability) was above 80% even at the highest ALAN values (Online Resource 1, Figure S9). Excluding surveys with < 90% detection probability from our relative abundance model did not substantially change the coefficient estimates for any nightjar group (Online Resource 1, Figure S10), indicating the effect of ALAN on nightjar counts was not confounded by the slightly lower detection probability for surveys with higher ALAN. When ALAN increased from 0 nWcm^−2^sr^−1^ to the 95th percentile ALAN value (6.18 nWcm^−2^sr^−1^), detection probability did not change for extra-territorial (vocalizing) Common Nighthawks, decreased by 7% (− 13%, − 1%) from 95% (95%, 96%) to 89% (83%, 94%) for territorial Common Nighthawks, and by 5% (− 20%, + 1%) from 99% (99%, 99%) to 94% (80%, 100%) for Common Poorwills. These detection probabilities were calculated with sun angle, day of year, and lunar illumination held at their mean values. Even if detection probability at 6.18 nWcm^−2^sr^−1^ compared to unlit areas was reduced by 13% for territorial Common Nighthawks and 20% for Common Poorwills (the high ends of 95% credible intervals), reduced detection probability would account for only a small fraction for the reduction in the expected number of nightjars detected (territorial Common Nighthawks: 74% reduction (− 62%, − 83%), Common Poorwills: 83% reduction (− 97%, − 60%), assuming mean value for urban land cover).

## Discussion

As ALAN increases in both urban and remote areas, it potentially benefits species that hunt flying insects by aggregating their prey, but could also increase predation risk, especially for species that nest on the ground. We used data from the Canadian Nightjar Survey in British Columbia to test the foraging benefit and predation risk hypotheses by investigating whether the relative abundance of Common Nighthawks and Common Poorwills increased or decreased in areas with ALAN. For Common Nighthawks, we found that the association with ALAN depended on whether nighthawks were exhibiting territorial or extra-territorial behaviour, and on the level of urbanization. The increased relative abundance of extra-territorial Common Nighthawks in sites with ALAN supported the foraging benefit hypothesis, but only in areas with low proportions of urban land cover, and even in these areas uncertainty remains about the direction of the effect of ALAN due to large credible intervals around estimates. The predation risk hypothesis was supported by the decreased relative abundance of territorial Common Nighthawks and of Common Poorwills, which forage and nest within the same territory. Altogether, this work demonstrates that the effects of ALAN can shift depending on behavioural context, level of urbanization, and whether a species forages outside of its nesting territory.

### Foraging benefit hypothesis

Although many aerial insectivores have been observed foraging under artificial lights, our results suggest that these foraging benefits of ALAN may be limited to less urbanized areas and to species that can spatially separate their foraging from their nesting sites. We found a negative effect of artificial light on the relative abundance of Common Poorwills, suggesting that this species was not foraging under artificial lights. In contrast, the relative abundance of extra-territorial Common Nighthawks showed a positive association with artificial light in areas with low proportions of urban land cover, suggesting that ALAN was attracting them to areas with artificial light, presumably to forage. However, the lower bound of the 95% CI for the main effect of ALAN was 0, and the 95% CIs for the proportional change in relative abundance as ALAN increased included negative values at all levels of urban landcover (Online Resource 1, Table S4). The uncertainty about the association between relative abundance of extra-territorial Common Nighthawks and ALAN in our study area may indicate that the prevalence of this foraging behaviour varies across the diverse landscapes in the region. Further analysis of how their association with ALAN depends on land cover types could increase our understanding of where and how often Common Nighthawks benefit from foraging under artificial light.

The interaction between ALAN and urban land cover for extra-territorial Common Nighthawks resulted in a negative association in areas where urban land cover was > 18%, suggesting that they do not frequently forage under artificial lights in these areas. This level of urban land cover characterizes low-density neighborhoods and areas on the edges of towns and cities (Online Resource 1, Figure S11), so this negative relationship occurs even at low levels of urbanization. There are several possible explanations for why extra-territorial Common Nighthawks showed a negative relationship with ALAN in urban areas. The cumulative effects of urban stressors may reduce insect populations (Langevelde et al. 2018; Boyes et al. 2020, 2021), which could result in fewer insects attracted to streetlights (Camacho et al. 2021). Aerial-hawking bats have also been found to benefit more from artificial light in natural areas than in cities (Barré et al. 2021). Difficulty foraging in areas with urban clutter (e.g. fences and buildings), which prevented large-sized bats from foraging under ALAN (Li and Wilkins 2022), could also explain this pattern for nighthawks. Our simulations showed that our model had limited power to detect a positive main effect of urban land cover when we used the fitted coefficient value (+ 0.83). We only measured urban land cover, which is based on impervious surfaces, but including more specific metrics of urbanization that are less correlated with ALAN may reveal which aspects of human development affect the relative abundance of aerial insectivores additively or in interaction with ALAN.

Our final prediction for this hypothesis was not supported; the relative abundance of territorial nighthawks did not show a positive relationship with ALAN at the landscape scale as we expected if they traveled from their nest sites to forage under artificial lights. Despite fitting additional versions of our model to test for the marginal effects of landscape scale ALAN on the relative abundance of territorial nighthawks, we consistently found a negative effect. The discrepancy between the relative abundance of nesting and foraging nighthawks in light-polluted landscapes has several possible explanations.

### Predation cost hypothesis

Both territorial Common Nighthawks and Common Poorwills were negatively associated with ALAN, supporting the hypothesis that artificial light increases nest predation risk. Predation was the most common cause of nest failure in several studies of nightjars (Langston et al. 2007; Allen and Peters 2012). Nightjar eggs, nestlings, and incubating adults are particularly vulnerable to predators because they have limited mobility for three weeks after hatching (Brigham et al. 2020). Foraging adults have a lower predation risk because they can move away from predators, and artificial light may actually improve their ability to detect predators and take evasive action (Prugh and Golden 2014). The decrease in relative abundance of Common Nighthawks only when on their territories, where they are most vulnerable to predation, supports the hypothesis that increased predation risk drives this pattern of relative abundance. However, other stressors specific to nesting, such as ALAN’s impacts on sleep and nestling development (Raap et al. 2016; Grunst et al. 2020), could explain this pattern. The overall negative effect of ALAN on the relative abundance of Common Poorwills suggests that the costs of nesting near ALAN outweigh the foraging benefits for species that conduct both activities within one territory.

The response of Common Nighthawks and Common Poorwills to intermediate scale ALAN demonstrates how the impacts of ALAN extend far beyond directly illuminated areas, but our ability to identify the most predictive scale was limited. A spatial scale between 1.6 km and the next buffer size we tested (6.4 km) may have been selected if we could have included more scales in the BLISS model. The ALAN measured in the 1.6 km buffer included light that originated outside of that buffer because the EOG radiance estimates are influence by skyglow (Sanchez de Miguel et al. 2020). Furthermore, the radiance value for each pixel in the composite is influenced by light sources outside of the pixel boundary because the composites use area-weighted-averages of multiple images with different pixel positions and orientations (Kyba et al. 2020). While the spatial scale of ALAN’s impacts on the relative abundance of territorial nightjars is uncertain, it is likely larger than 1.6 km.

Artificial light may have affected nest predation risk directly by increasing skyglow or indirectly by affecting trophic relationships. Skyglow can increase ambient illumination levels tens of kilometers from a light source, especially on cloudy nights (Kyba et al. 2011; Jechow et al. 2017), which may have increased the actual or perceived nest predation risk for nightjars in our study area. Some nest predators like American Crows (*Corvus brachyrhynchos*) prefer to roost in illuminated areas at night (Gorenzel and Salmon 1995), which may also increase their abundance in artificially illuminated landscapes during the day. Our results contrast with studies that found no correlation between breeding bird densities and ALAN when studying non-ground nesting species and only measuring ALAN at a local scale (Jong et al. 2015; Russ et al. 2017; Wang et al. 2021). This contrast suggests that ALAN affects ground-nesting nocturnal birds more than other species, or that the effects of ALAN occur at larger spatial scales than measured in other studies.

Common Nighthawks can forage far from their nest sites, possibly allowing them to reap the benefits of foraging on insects that aggregate under ALAN while avoiding any negative impacts of lighting on nest success. However, the negative effects of ALAN on territorial Common Nighthawks across multiple spatial scales casts doubt on whether individuals with territories are actually foraging under ALAN. There are several possible explanations for the lower relative abundance of territorial nighthawks in light polluted landscapes despite the higher relative abundance of extra-territorial individuals. Nesting nighthawks may have traveled farther than 6.4 km to forage under artificial lights, which would require a high energetic benefit from this foraging behaviour to sustain the travel cost (Evens et al. 2018). Another explanation is that there were more nighthawks nesting in artificially lit landscapes than we counted, but they spent less time wingbooming because nestlings with increased nocturnal activity under ALAN required the adults to spend more time foraging to meet their energetic demand (Titulaer et al. 2012, but see Welbers et al. 2017 and Injaian et al. 2021). Alternatively, most individuals foraging under artificial lights may not have been able to establish a nest or they made breeding attempts that failed (Van Horne 1983). Because the Canadian Nightjar Survey does not track individuals over time or conduct repeat visits, we cannot evaluate these potential explanations. Our analysis shows that the impacts of ALAN on patterns of nightjar relative abundance are widespread, and the processes that drive these patterns occur throughout the species’ ranges in British Columbia.

### Implications

Reports of species foraging under artificial lights in particular locations should not be interpreted to mean that this behaviour is ubiquitous and that ALAN has net benefits for them. Our results contrast with reports of nightjars sometimes foraging under artificial light in cities (Shields and Bildstein 1979; Foley and Wszola 2017). Occasional observations of foraging under ALAN do not necessarily mean that this behaviour is common in a population or species relative to individuals of the same population foraging in less illuminated areas. Due to these observation biases, this behaviour may be overrepresented in the literature. Research that covers large spatial extents and includes both illuminated and unilluminated areas is important for understanding whether this behaviour is widespread enough to impact species abundance patterns. Community science programs should continue to target dusk and nighttime surveys, documenting all bird species seen or heard, to better understand the impacts of ALAN over broad spatial scales.

Evaluating the effects of ALAN using community science programs requires special considerations. In most study areas, roadside surveys likely sample developed and artificially lit areas at higher proportions than their availability. This sample bias could help achieve the sample sizes necessary to evaluate the effects of ALAN on the occurrence or abundance of bird species, but they would likely overestimate the overall exposure of target species to ALAN. Detections of each individual should be recorded during each survey minute in order to evaluate the effect of ALAN on detection probability because ALAN can affect signal production rate (Dickerson et al. 2022, 2023, Nakamura-Garcia and Ríos-Chelén 2021). By modeling the detection probability of an individual in each 6-min survey, we were able to confirm that the reduction in the number of nightjars observed in areas with artificial light could not be explained changes to detection probability. However, our detection probability estimates for territorial Common Nighthawks and Common Poorwills may be unreliable at high ALAN values because data were sparse: only 10 territorial Common Nighthawks and 3 Common Poorwills were detected during surveys with ALAN radiance > 3 nWcm^−2^sr^−1^. Targeted observations of nightjars in areas with high ALAN radiance could confirm that detection probability in a 6-min survey remains high.

Behavioural research over smaller spatial scales is also necessary to reveal the mechanisms that drive the patterns we observed in our study. Experimental illumination over several breeding seasons could reveal whether the introduction of ALAN alters relative abundance of ground-nesting species and foraging aerial insectivores, and how it affects their survival and reproductive success. Foraging under artificial lights may result in lower survival and/or reproductive success if it exposes nightjars and other birds to road mortality, especially if they roost on gravel roads between foraging bouts (Jackson and Slotow 2002; Jackson 2003; Fortney 2010). Birds preying on insects could themselves become prey to raptors whose hunting activity extends into the night in artificially lit areas (Rutz 2006; Canário et al. 2012; Buij and Gschweng 2017). Ultimately, experimental and mechanistic studies are needed to understand how ALAN’s influence on behaviour and habitat use influences population trajectories for aerial insectivores and/or ground-nesting birds.

Our results suggest that limiting light pollution in the ranges where nightjars occur would have positive effects on these species. Efforts to reduce the impacts of ALAN for nightjars could target nest sites identified by the Canadian Nightjar Survey and eBird, as well as likely nest sites, which include gravel, sand, bare rock, recently disturbed forest, and open pine forest (Brigham et al. 2020; Knight et al. 2021b). Because the impacts of ALAN extend beyond the directly illuminated area, nightjars could benefit from reduced artificial light within several kilometers of ecologically sensitive areas.

Highlighting ALAN’s impacts on breeding birds of sensitive and declining species could increase public support for reducing light pollution during the breeding season, just as bird collisions with illuminated structures have inspired efforts to turn off city lights during migration (National Audubon Society). Surveys have found that the negative effects of ALAN on wildlife motivate people to support light pollution regulation (Lyytimäki and Rinne 2013; Beaudet et al. 2022). Reducing light pollution during the avian breeding season would benefit other taxa, including insects, bats, and even humans (Svechkina et al. 2020).

There are many strategies for reducing light pollution, including removing unnecessary light sources and preventing new light sources from being installed (Gaston et al. 2012). When lights cannot be eliminated, motion sensors can turn them on only when light is needed and dimming lights can reduce their ecological impacts (Rowse et al. 2018). Shading light sources can limit the directly illuminated area and reduce skyglow, limiting the spatial extent of ALAN’s impacts. As humans extend our activities into the night over a growing portion of the globe, year-round reductions in light pollution will promote both human and ecological health.

### Supplementary Information

Below is the link to the electronic supplementary material.

Supplementary file1 (PDF 3344 kb)

## Data Availability

Data from the Canadian Nightjar Survey are available through the NatureCounts platform (https://naturecounts.ca/nc/default/main.jsp). To request a copy of the data organized into number of individuals counted per survey for Common Nighthawks and Common Poorwills, please contact caadams1@colostate.edu.
