# Supplementary material for: Behaviour and landscape contexts determine the effects of artificial light on two crepuscular bird species
Source: Landsc Ecol. 2024 Mar 26;39(4):83. doi: 10.1007/s10980-024-01875-3 (PMC10965584; doi:10.1007/s10980-024-01875-3)

Supplemental Tables and Figures

Figure S1 Comparison of artificial light estimates in NASA's Black Marble (BM) annual composite for 2015 to the Earth Observation Group's annual composite for 2015 version 1 (V1). Blue areas show pixels assigned positive artificial light values by BM, but not by V1. Red areas show pixels assigned positive artificial light values by V1, but not by BM. Visual inspection showed that the red areas occurred along roadways and small communities, while blue areas more often occurred at high elevations or latitudes.

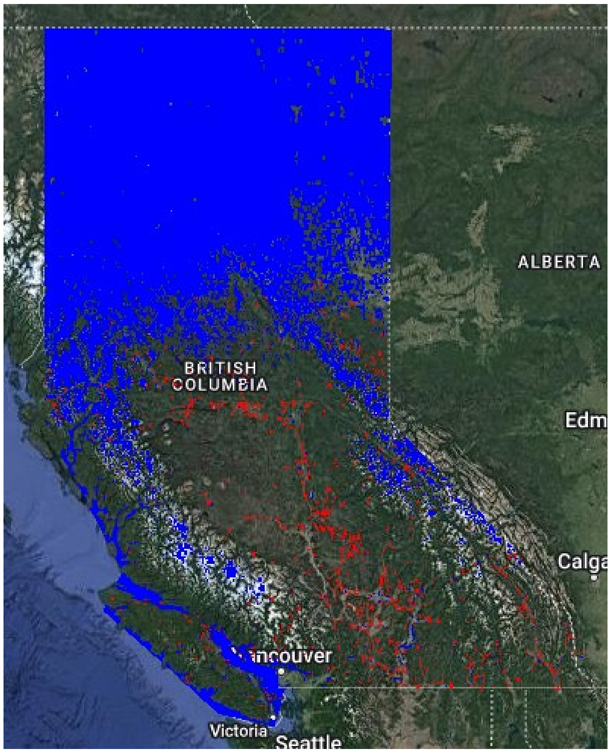

Figure S2 Comparison of the Earth Observation Group's Version 1 (V1) and Version 2 V(2) annual composites for 2015**.**

Red areas show pixels assigned positive artificial light values by V1, but not by V2. Blue areas show pixels assigned positive artificial light values by V2, but not by V1. Visual inspection showed that blue areas (missed by V1) likely involved skyglow in the pixels surrounding artificial light sources. The red pixels (missed by V2) occurred in areas with low levels of human development where V1 identified light sources that V2 missed. V1 is available for only 2015 and 2016, and uses a combination of automated and manual processes for distinguishing artificial light from aurora in the Northern aurora zone (Elvidge et al. 2017). V2 is available for all years between 2012 and 2020, but applies an additional manual filter before manual editing in the Northern aurora zone, filtering out many dim lights in our study area that were found in V1.

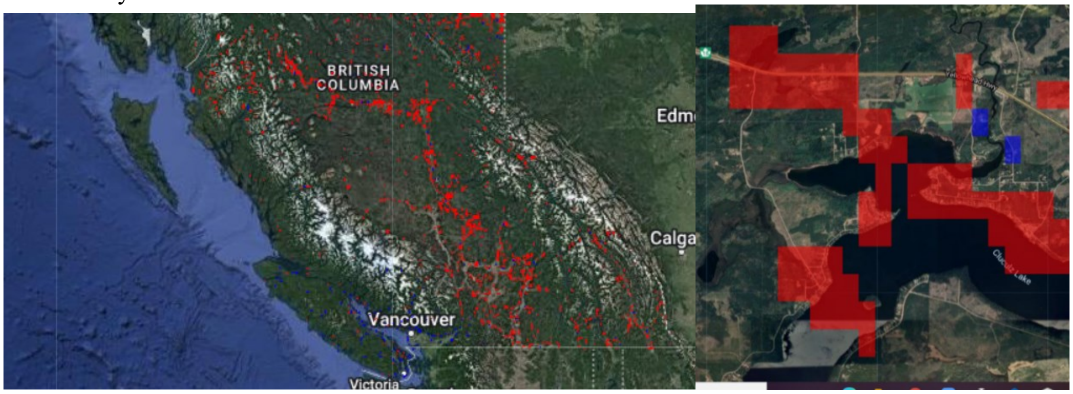

Table S1 Common Nighthawk associations with landscape features in previous studies

Species/Reference / Location	Landscape feature	Method of measurement
**Common Nighthawk / Ng 2008 /** Saskatchewan grassland	Percentage grassland cover (+)<br>Distance to nearest water (-)<br>NDVI (-)<br>Roost on gravel roads	Generalised Landcover for the Canadian Prairies from the Prairie Farm Rehabilitation Administration
**Common Nighthawk /** Knight, Brigham, and Bayne 2021 / Alberta Boreal Forest	Time since harvest (-)<br>Time since well pad abandonment (-)<br>Time since fire (-)<br>% Open pine forest (+)<br>Mean wetland probability (quadratic)	ABMI Human footprint inventory (time since harvest and well-site)<br>Alberta Agriculture and Forestry Historical Wildlife Perimiters<br>Alberta Vegetation Inventory (Proportion Pine Forest)<br>Boreal Wetland Probability
**Common Nighthawk /** Viel 2020 / Urban and agricultural areas in Wisconsin	% Agriculture (-)<br># of flat, graveled rooftops (+)	Aerial photos<br>National Land Cover Dataset
**Common Nighthawk /** Newberry 1997 / Agricultural landscape in South Dakota	Developed landcover (+) (but only in the area where gravel rooftops were present)<br>Agriculture (-) (but only in the area where there was no grassland left)	National Land Cover Dataset
**Common Nighthawk /** Newberry and Swanson 2018/ South Dakota, Nebraska, and Iowa	Agriculture(+/-) (negative overall but positive in one region)<br>Grassland (+)<br>Developed landcover (+) (but only in the area where gravel rooftops were present)	National Land Cover Dataset
**Common Nighthawk /** Farrell et al. 2019 / Ontario Boreal Forest	Open wetland (+)<br>Age of clearcut (-)	Forest resource inventory
**Common Nighthawk /** Farrell et al. 2017 / Ontario boreal forest	Burned stands (+)<br>Recent clearcuts (+)<br>Open wetlands (+)	Fire maps from Ontario Ministry of Natural Resources and Fire Maps
**Common Poorwill / MacDonald, David, and McMaster 2003 /** Southwest Saskatchewan	Native prairie (+)<br>Native rangeland (+) with sandy soil or shrubby vegetation	Saskatchewan's Southern Digital Landcover Classification

Table S2 DIC comparison for alternative model forms

	**Model Form**	**DIC**
Territorial Common Nighthawks	**Negative binomial**	**7137.457**
	Poisson	7975.703
	Zero-inflated negative binomial	*Does not converge*
	Zero-inflated Poisson	9092.844
Extra-territorial Common Nighthawks	**Negative Binomial**	**10246.62**
	Poisson	11115.01
	Zero-inflated Negative Binomial	*Does not converge*
	Zero-inflated Poisson	13585.5
Common Poorwills	**Negative binomial**	**2015.274**
	Poisson	2258.389
	Zero-inflated Negative Binomial	2360.337
	Zero-inflated Poisson	2097.452

Bold values are the lowest DIC model for each analysis.

Figure S3 Coefficient estimates from model fit with simulation data

We simulated relative abundance data using the coefficient values and scales selected by our model. These blue posterior density plots show the coefficient estimates from models fit using these simulated relative abundance values, the mean estimates (solid lines), and 95% credible interval (dashed lines). The pink lines show the coefficient value used for the simulation. To estimate the coefficients from the simulated data, we used the same process that we used for the real data. We used the BLISS procedure to select the scale for each covariate, then we refit the model with each covariate at its selected scale. For all models, the BLISS model correctly selected the ALAN or urban scale that was used to simulate the data, except in the case of Grassland of Territorial Common Nighthawks, where the model fit with simulated data selected 400 m instead of 1600 m.

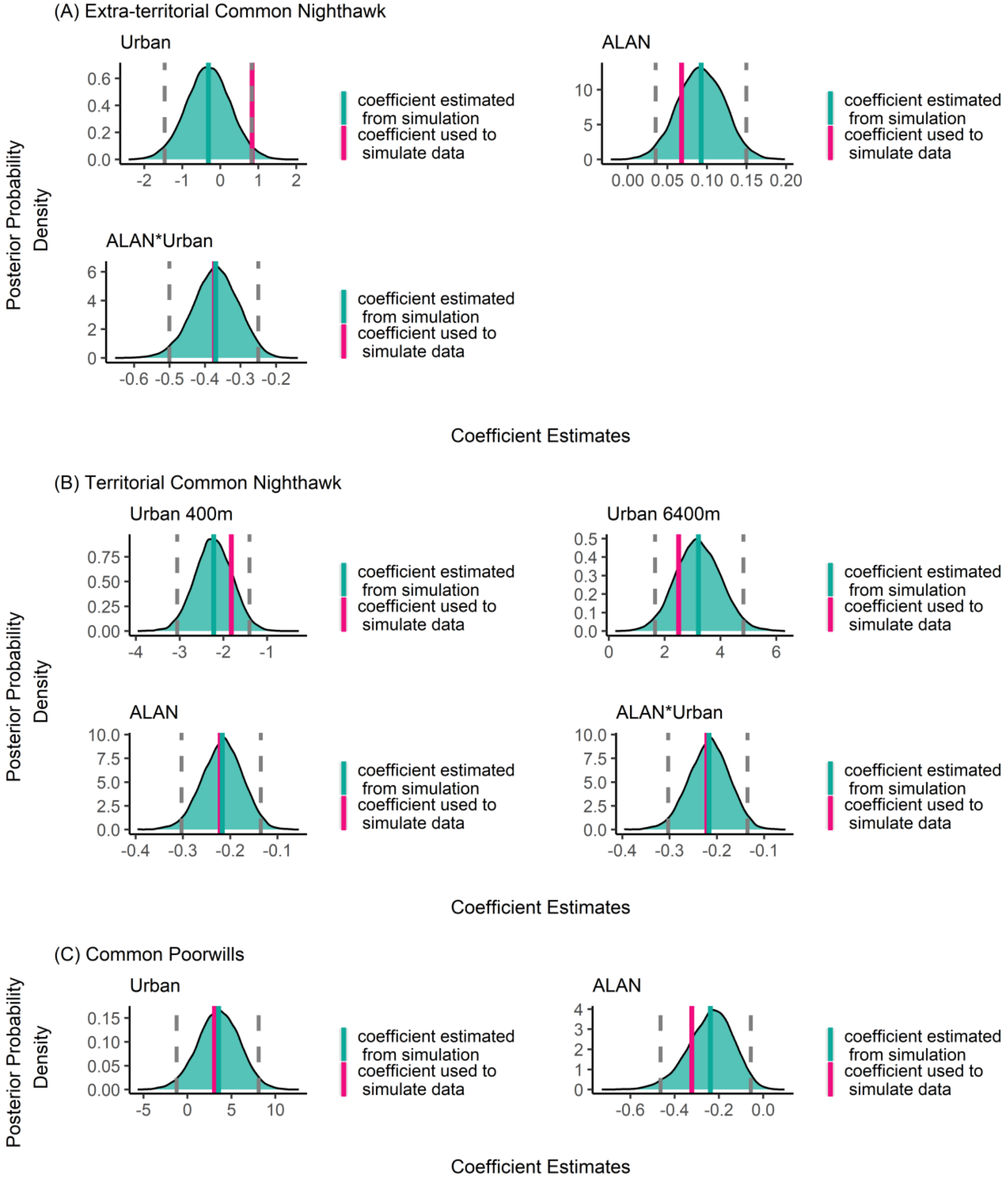

Table S3 Scale(s) selected for each landscape covariate and the proportion of the posterior distribution that selected these scales.

**Modelling Step**	**Predictor**	**Extra-territorial Common Nighthawks***		**Territorial Common Nighthawks**		**Territorial Common Nighthawks (without Victoria points)**	
		Selected scale(s)	Prop. of posterior	Selected scale(s)	Prop. of posterior	Selected scale(s)	Prop. of posterior
**1 (scale selection on all covariates)**	**Agriculture**	6400	100%	1600	94%	1600	86%
	**Grassland**	1600	96%	1600	42%	1600	40%
	**Shrub**	6400	100%	6400	100%	6400	100%
	**Sparse Tree**	1600	100%	400	93%	400	93%
	**Water**	1600	99%	1600	88%	1600	66%
**2 (scale selection on ALAN and Urban only)**	**Urban**	6400	52%	400(-), 6400(+)	89%, 7%	400	90%
	**ALAN**	6400	96%	1600	100%	1600	97%
	**ALAN*Urban**	6400* 1600	98%*91%	6400*6400	92%*93%	1600*1600	84%*62%

**Modelling Step**	**Predictor**	**Common Poorwills (with interaction)**		**Common Poorwills (no interaction)**	
		Selected scale(s)	Prop. of posterior	Selected scale(s)	Prop. of posterior
**1 (scale selection on all covariates)**	**Agriculture**	1600	100%	1600	100%
	**Grassland**	1600	55%	1600	53%
	**Shrub**	6400(+), 400(-)	29%, 43%	6400(+), 400(-)	27%, 46%
	**Sparse Tree**	1600	53%	1600	51%
	**Water**	1600	71%	1600	68%
**2 (scale selection on ALAN and Urban only)**	**Urban**	6400	63%	6400	54%
	**ALAN**	1600	56%	1600	61%
		6400*6400	42%*38%		

Figure S4 Posterior probability densities for ALAN and urban landcover covariates in the sensitivity analysis for the version of the annual composite used to estimate ALAN.

The solid lines show the mean coefficient estimate from model fit using each version of the annual composite and the dashed lines show the 95% CIs. V1 is the Earth Observation Group (EOG) Annual Composite V1 for 2016, V2 is the EOG Annual Composite V2 for the year in which the survey took place, and V1V2 is the average of these two composites.

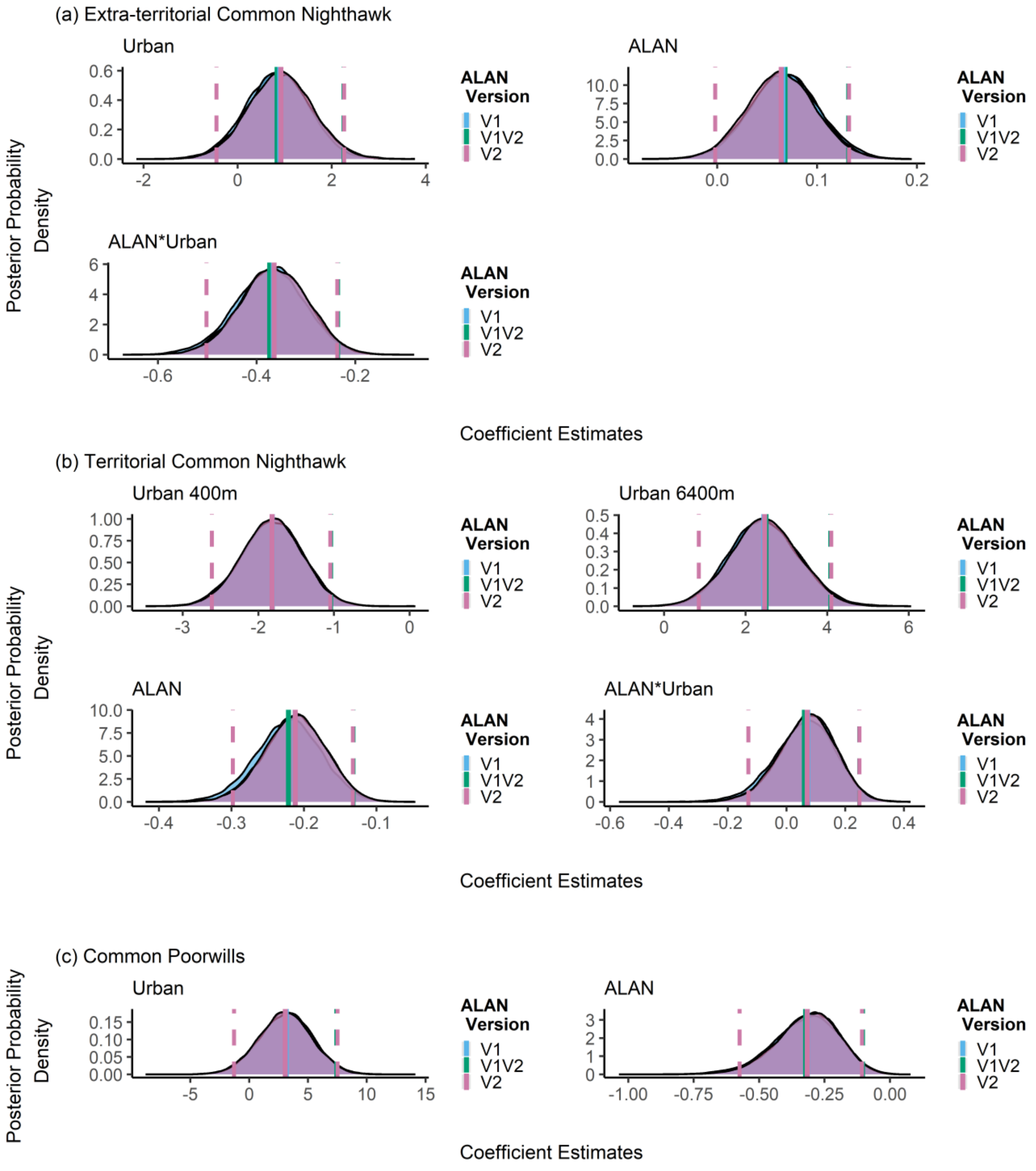

Figure S5 Results from BLISS scale selection

The left side of the figure shows the proportion of the posterior selecting each scale. The right side of the figure shows boxplots of the coefficient estimates from the portions of the posterior distributions that selected each scale.

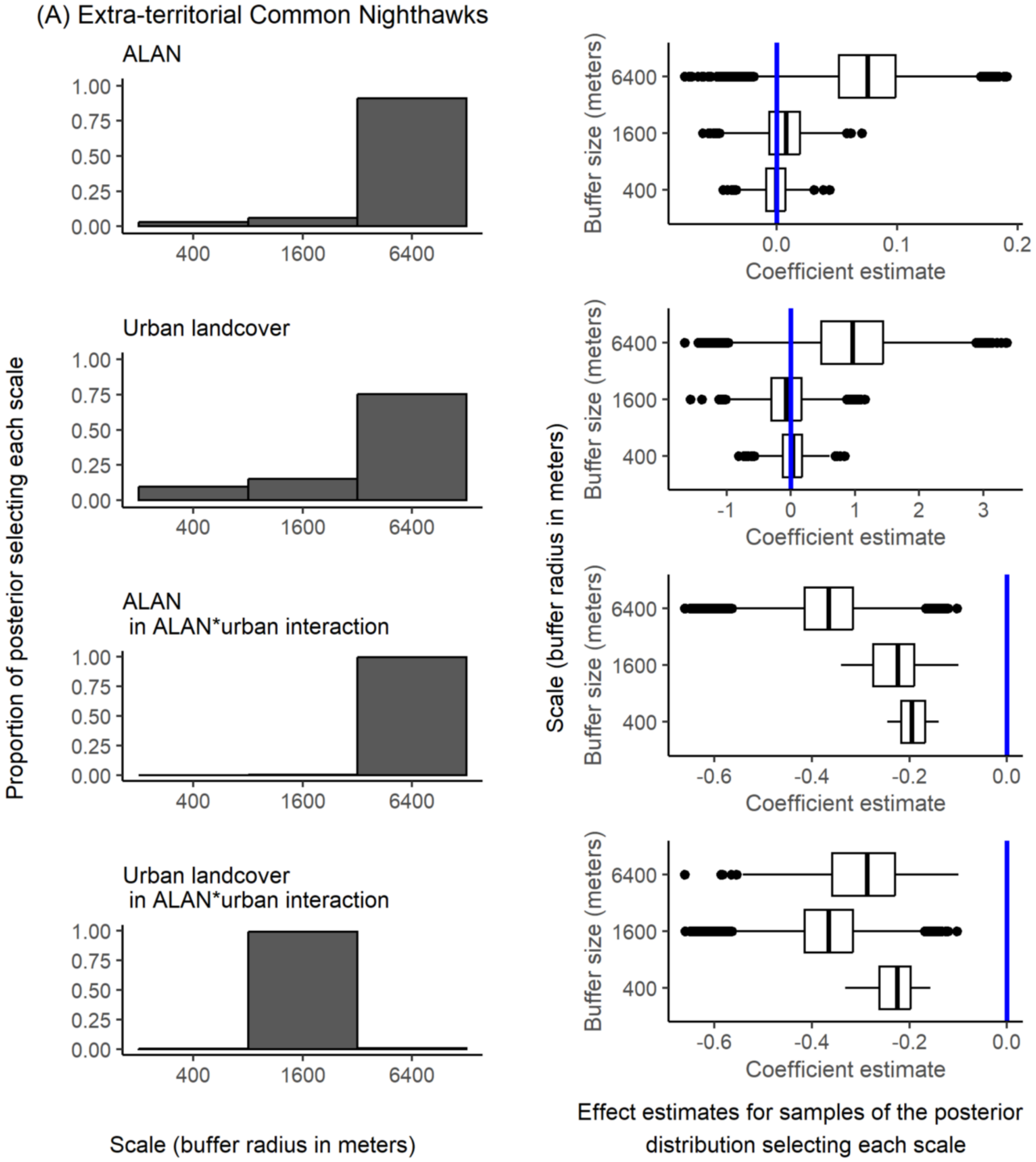

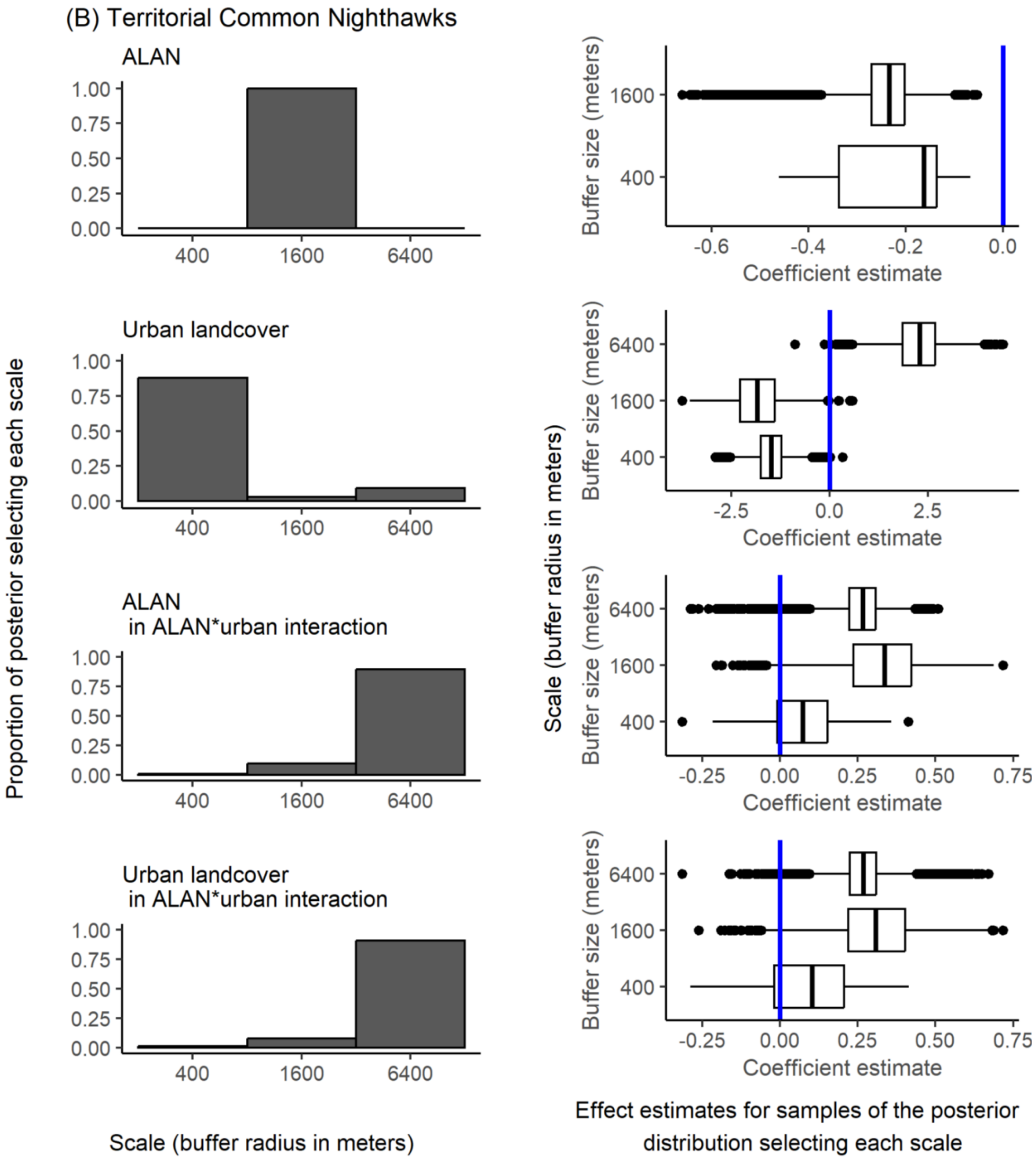

(B) Territorial Common Nighthawks
ALAN
Urban landcover
ALAN
in ALAN*urban interaction
Urban landcover
in ALAN*urban interaction
Proportion of posterior selecting each scale
1.00
0.75
0.50
0.25
0.00
400
1600
6400
Scale (buffer radius in meters)
Buffer size (meters)
Coefficient estimate
-0.6
-0.4
-0.2
0.0
-2.5
2.5
-0.25
0.00
0.25
0.50
0.75
Scale (buffer radius in meters)
Effect estimates for samples of the posterior distribution selecting each scale

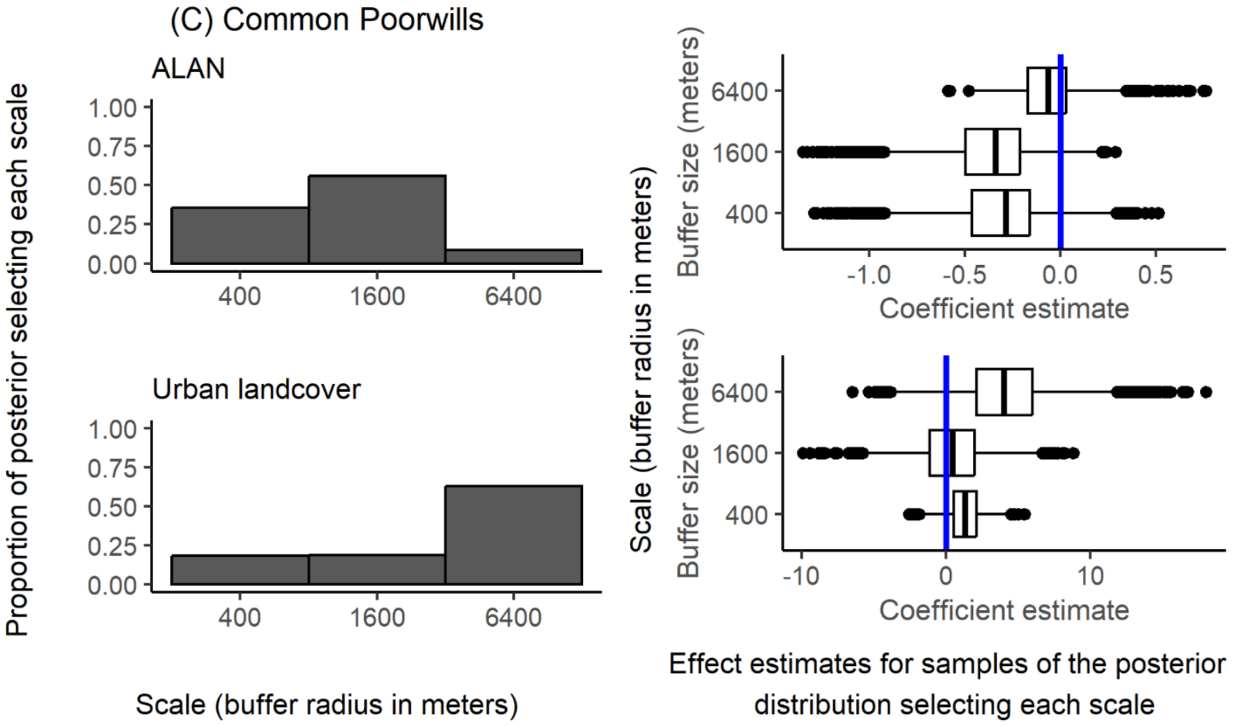

(C) Common Poorwills
ALAN
Urban landcover
Proportion of posterior selecting each scale
1.00
0.75
0.50
0.25
0.00
400
1600
6400
Scale (buffer radius in meters)
Buffer size (meters)
-1.0
-0.5
0.0
0.5
-10
0
10
Coefficient estimate
Scale (buffer radius in meters)
Effect estimates for samples of the posterior distribution selecting each scale

Figure S6 Posterior probability densities for landscapes covariates

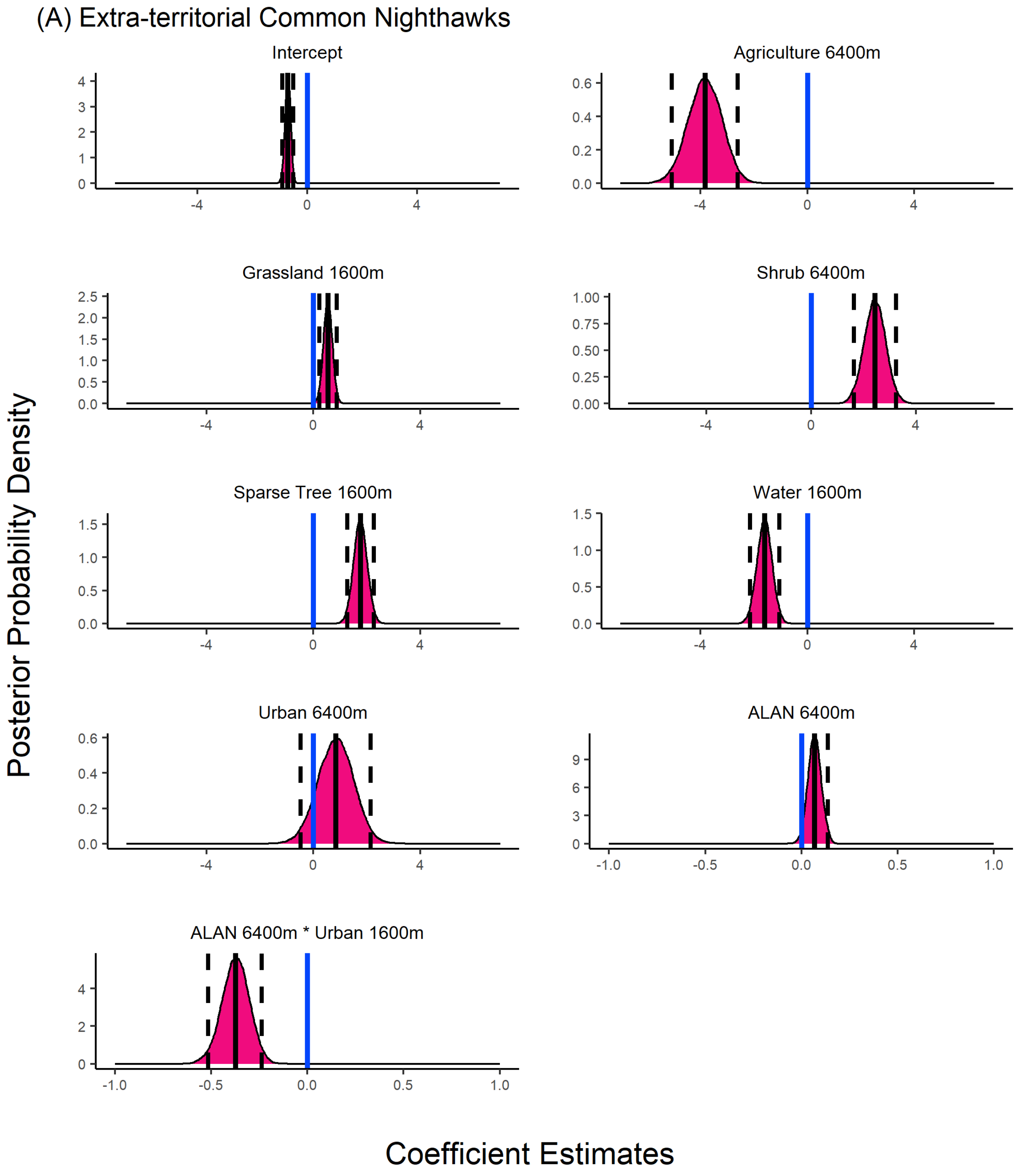

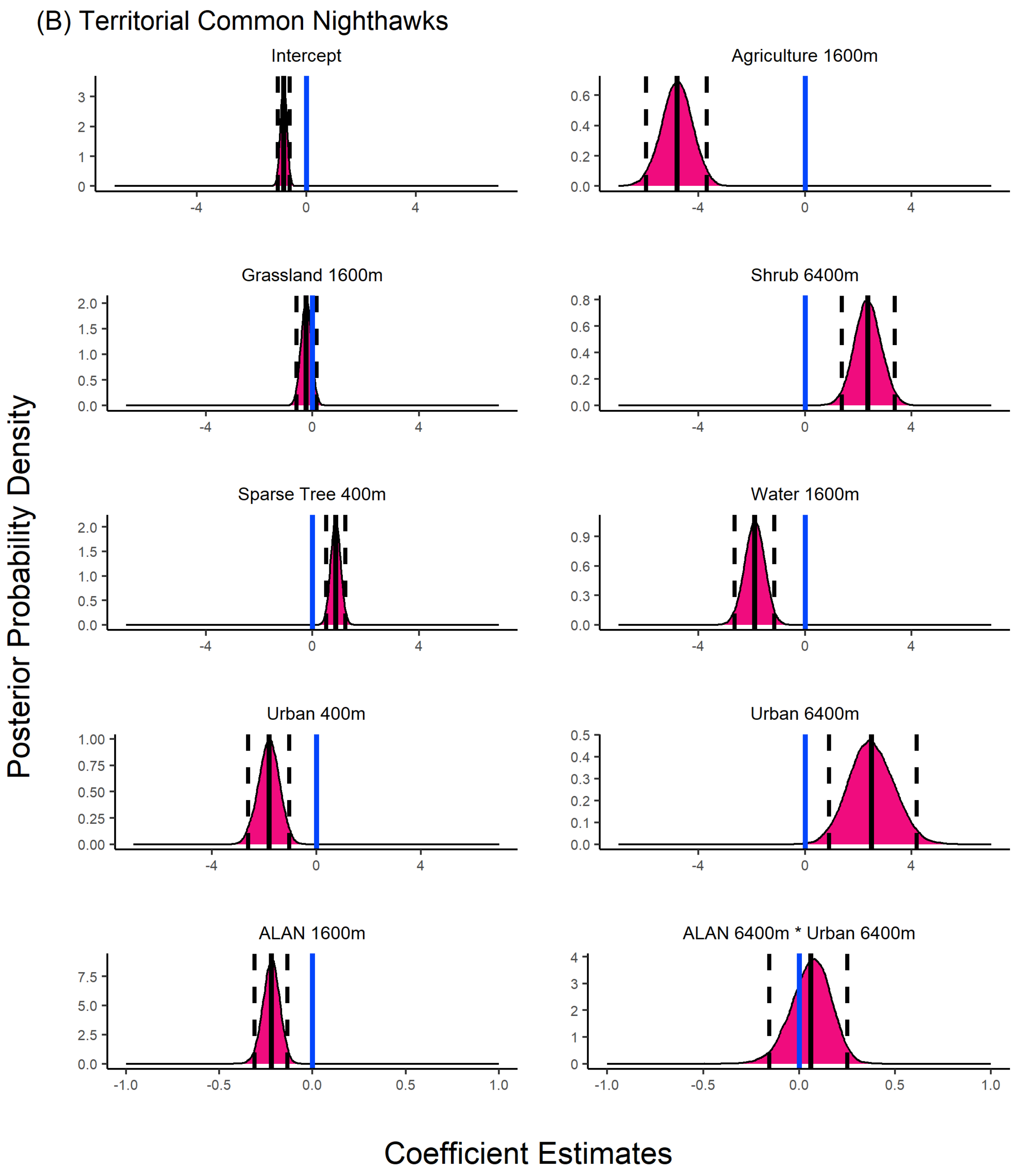
(B) Territorial Common Nighthawks
Intercept
Agriculture 1600m
Grassland 1600m
Shrub 6400m
Sparse Tree 400m
Water 1600m
Urban 400m
Urban 6400m
ALAN 1600m
ALAN 6400m * Urban 6400m
Posterior Probability Density
Coefficient Estimates

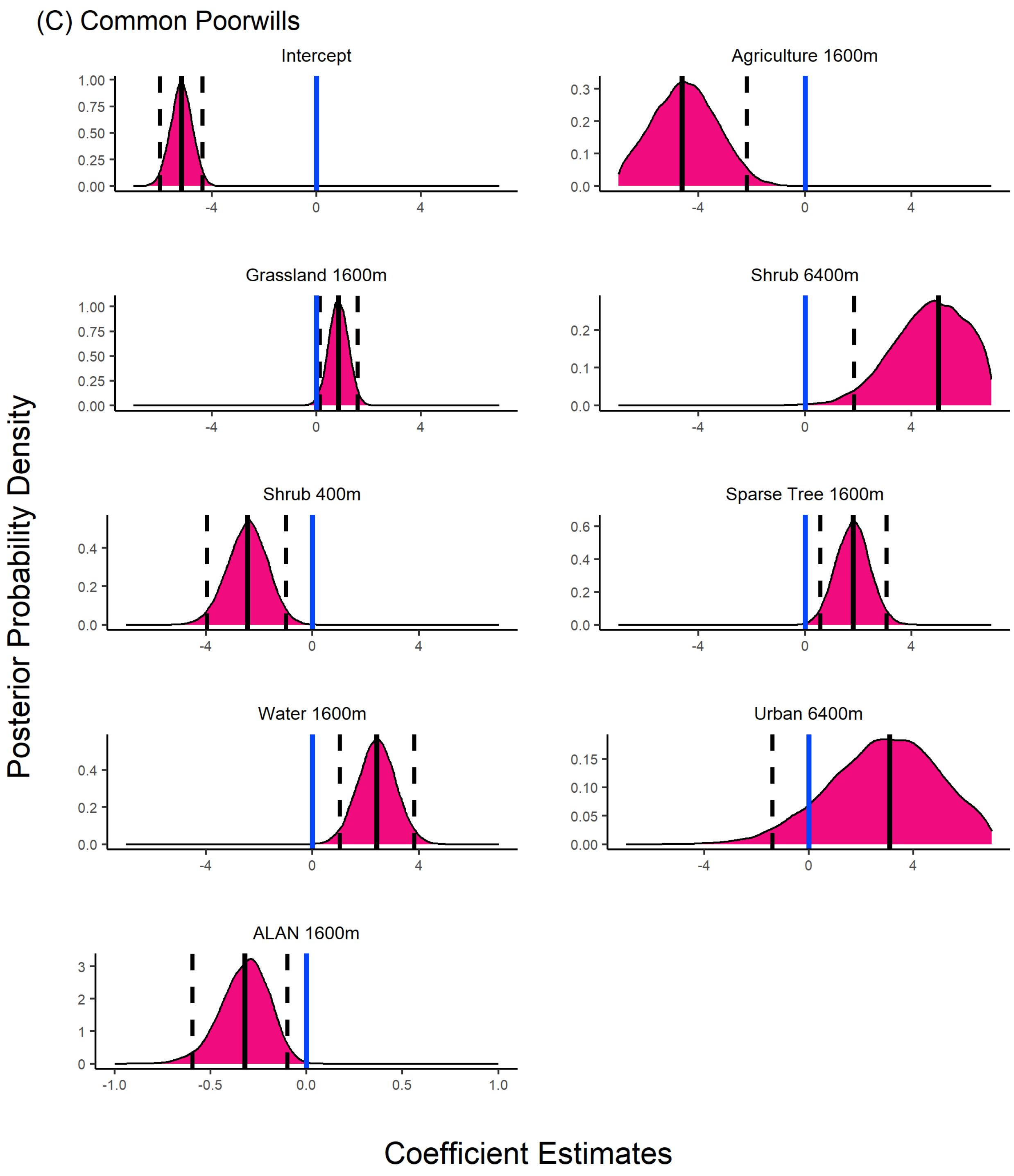
(C) Common Poorwills
Intercept
Agriculture 1600m
Grassland 1600m
Shrub 6400m
Shrub 400m
Sparse Tree 1600m
Water 1600m
Urban 6400m
ALAN 1600m
Posterior Probability Density
Coefficient Estimates

Figure S7 Posterior probability densities in the model for territorial Common Nighthawks excluding four influential survey stations near Victoria

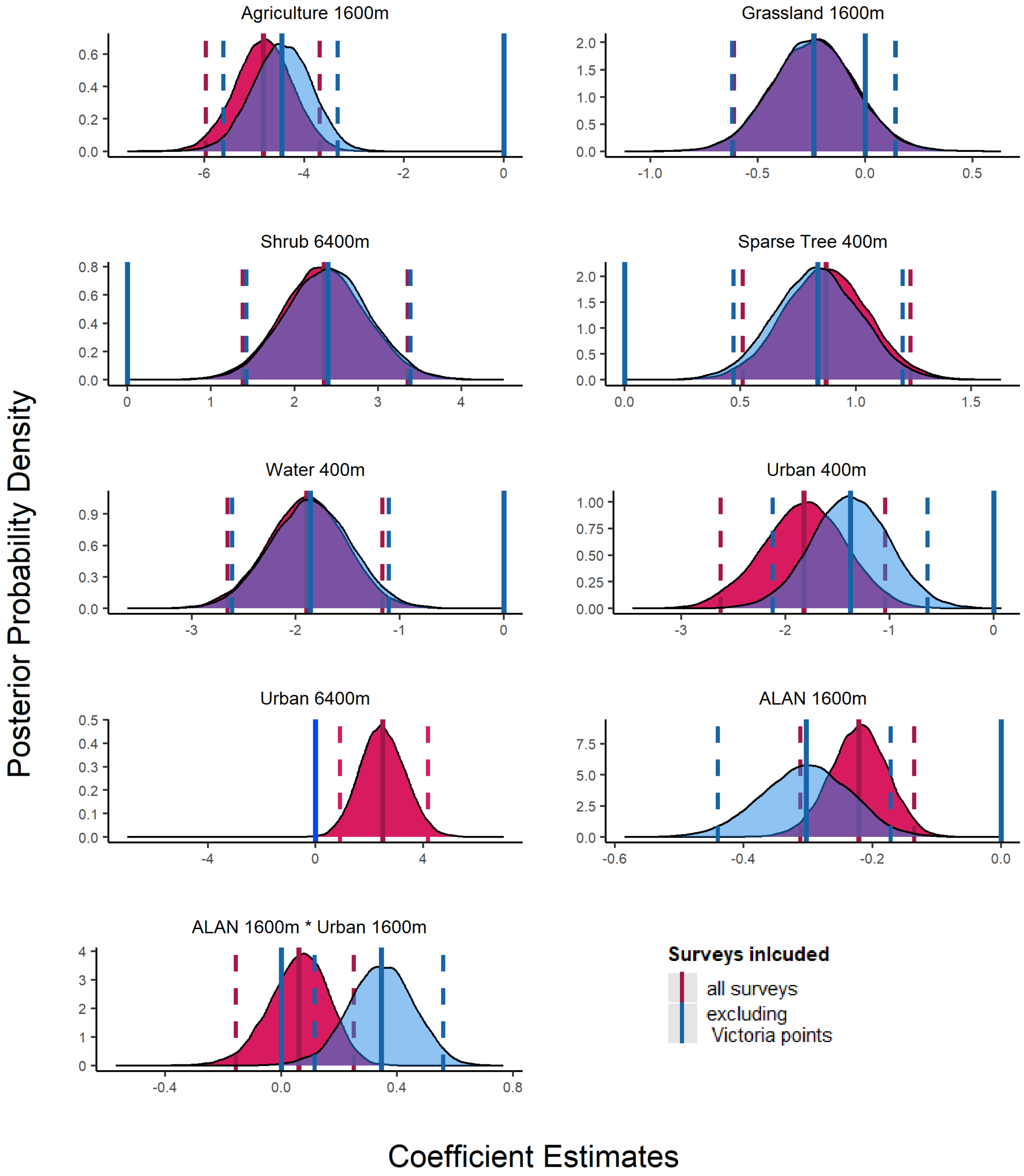

Table S4 Estimated effect of increasing ALAN on relative abundance of nightjars at mean, median, and high levels of urban landcover

Columns 1 and 2 show the coefficient estimates for ALAN and the interaction between urban landcover and ALAN in each model. The posterior distributions of the effect coefficients for other covariates are shown in Figure S5. Columns 3-5 show the expected change and the 95% CI for the change in the number of nightjars when ALAN increased from 0 to the 99th percentile. This 99th percentile ALAN value (shown in italics) was calculated for model and urban landcover level separately, using surveys with urban landcover less than or equal to the median, mean, and high (95th percentile) urban landcover values within the buffer size selected for each model.

	**Effect coefficient estimate (95% CI) for ALAN**	**Effect coefficient estimate (95% CI) for ALAN interaction with urban landcover**	**Change in nightjar counts between 0 $nWcm^{-2}sr^{-1}$ and *99th percentile ALAN value***		
			**Median urban landcover**	**Mean urban landcover**	**High urban landcover**
**Extra-territorial Common Nighthawks**	0.07<br>(0.00, 0.14)	-0.37<br>(-0.52, -0.23)	+25%<br>(-4% , +61%)<br>*3.98 $nWcm^{-2}sr^{-1}$*	+18%<br>(-14%, +60%)<br>*5.20 $nWcm^{-2}sr^{-1}$*	-59%<br>(-78%, -30%)<br>*10.30 $nWcm^{-2}sr^{-1}$*
**Territorial Common Nighthawks**	-0.22<br>(-0.31, -0.14)	0.06<br>(-0.16, 0.25)	-27%<br>(-36%, -18%)<br>*1.44 $nWcm^{-2}sr^{-1}$*	-56%<br>(-69%, -40%)<br>*3.76 $nWcm^{-2}sr^{-1}$*	-88%<br>(-96%, -74%)<br>*9.98 $nWcm^{-2}sr^{-1}$*
**Territorial Common Nighthawks (not including Victoria points)**	-0.30<br>(-0.44, -0.17)	0.34<br>(0.11, 0.56)	-33%<br>(-45%, - 21%)<br>*1.44 $nWcm^{-2}sr^{-1}$*	-60%<br>(-75%, -42%)<br>*3.76 $nWcm^{-2}sr^{-1}$*	-77%<br>(-89%, -60%)<br>*3.76 $nWcm^{-2}sr^{-1}$*
**Common Poorwills**	-0.32<br>(-0.60, -0.10)	*NA*	-32%<br>(-52% , -12%)<br>*1.23 $nWcm^{-2}sr^{-1}$*	-59%<br>(-83%, -26%)<br>*3.00 $nWcm^{-2}sr^{-1}$*	-84%<br>(-98%, -48%)<br>*6.55 $nWcm^{-2}sr^{-1}$*

Table S5 Marginal effects of landscape-scale ALAN on nesting nighthawks

**Scale**	**Mean coefficient estimate (95% CI)**
**6400 m, in model including both 1600 m and 6400 m buffers**	-0.07 (-0.168, 0.036)
**6400 m, in model only including surveys at stations with no ALAN in 1600 m buffer**	0.06 (-27.810, 0.009)

Figure S8 Cross-correlation among coefficient estimates for ALAN, urban, and their interaction term

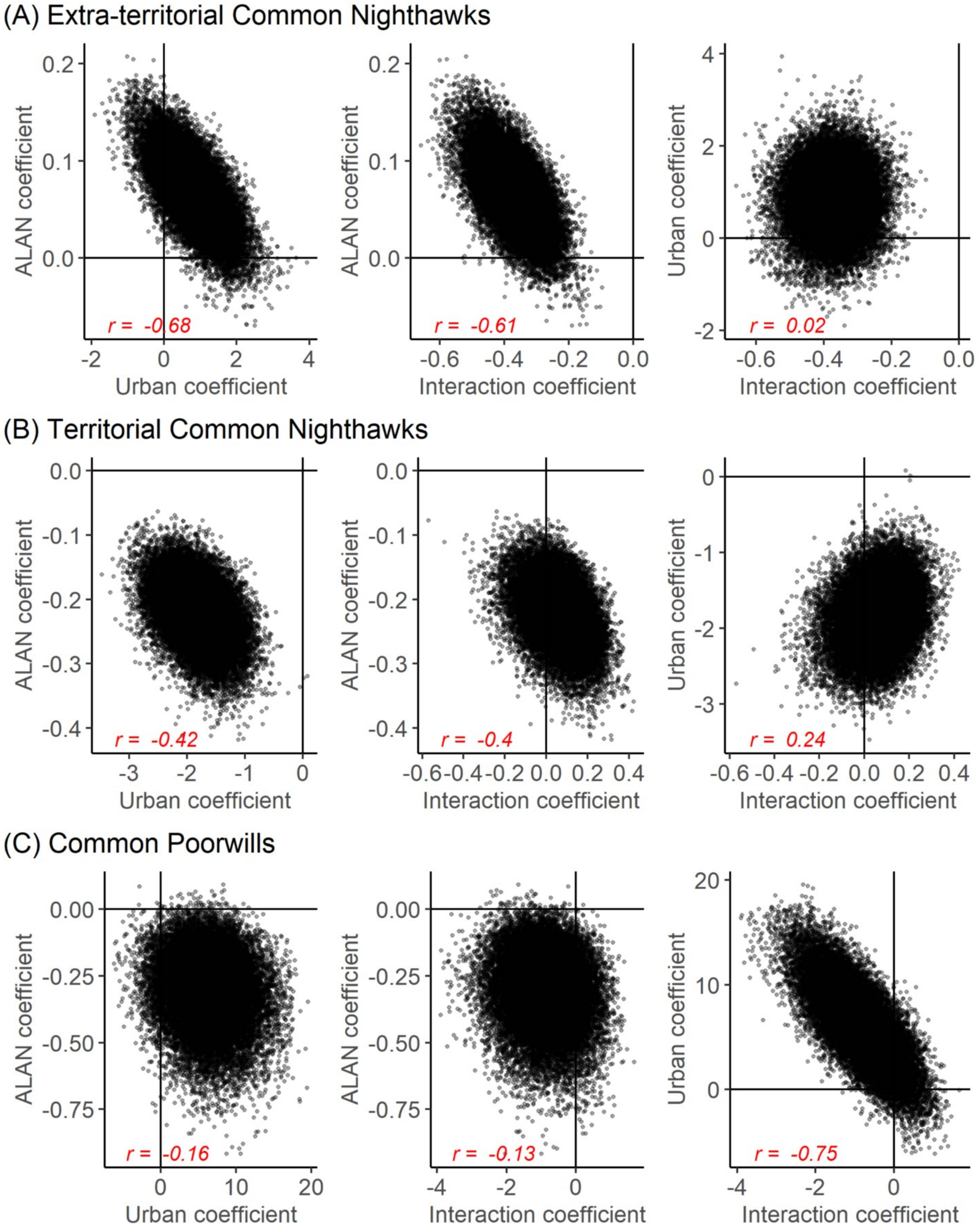

Table S6 Coefficient estimates from detection probability models

Using the minute-by-minute detection data for each individual nightjar, we modeled the effect of artificial light and temporal covariates on the number of minutes (out of six) in which each individual was detected using a binomial GLM. Bolded cells contain coefficients whose 95% CIs do not overlap zero.

	Extra-territorial Common Nighthawks	Territorial Common Nighthawks	Common Poorwill
ALAN	0.009 (-0.032, 0.053)	**-0.063 (-0.111, -0.019)**	-0.087 (-0.221, 0.035)
Sun Angle	-0.013 (-0.026, 0.000)	**-0.030 (-0.045, -0.016)**	**-0.042 (-0.080, -0.005)**
Sun Angle^2	**-0.002 (-0.003, -0.001)**	-0.001 (-0.003, 0.000)	-0.002 (-0.005, 0.002)
Julian Day	0.367 (0.231, 0.502)	0.084 (-0.048, 0.217)	0.098 (-0.007, 0.204)
Julian Day^2	**-0.001 (-0.001, -0.001)**	0.000 (-0.001, 0.000)	0.000 (-0.001, 0.000)
Lunar Illumination	**2.408 (1.066, 3.774)**	1.081 (-0.167, 2.319)	**3.795 (1.627, 5.951)**

Figure S9 Effects of (A) ALAN, (B) sun angle, and (C) Lunar illumination on detection probability in a 6-minute survey**.**

We modeled the detection rate for each individual as a function of artificial light and the temporal covariates, and made predictions across ALAN, sun angle, and lunar illumination values, with ordinal day at its mean value (178.2). Values on the x-axes span the 5th to 95th percentiles of ALAN, sun angle, and lunar illumination values observed across all surveys. Dashed lines represent the 5th and 95th percentile values observed in surveys where each nightjar species/behaviour occurred. The 5th percentile ALAN ad lunar illumination values for all species/behaviours were 0.

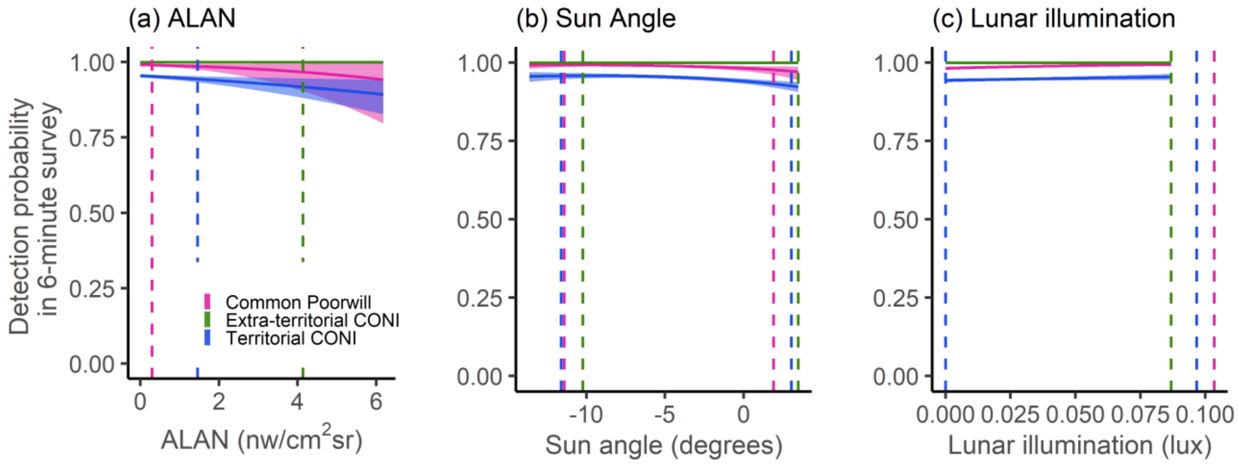

Figure S10 Posterior probability densities for ALAN and urban landcover coefficients in our sensitivity analysis for including/excluding surveys with less than 90% detection probability

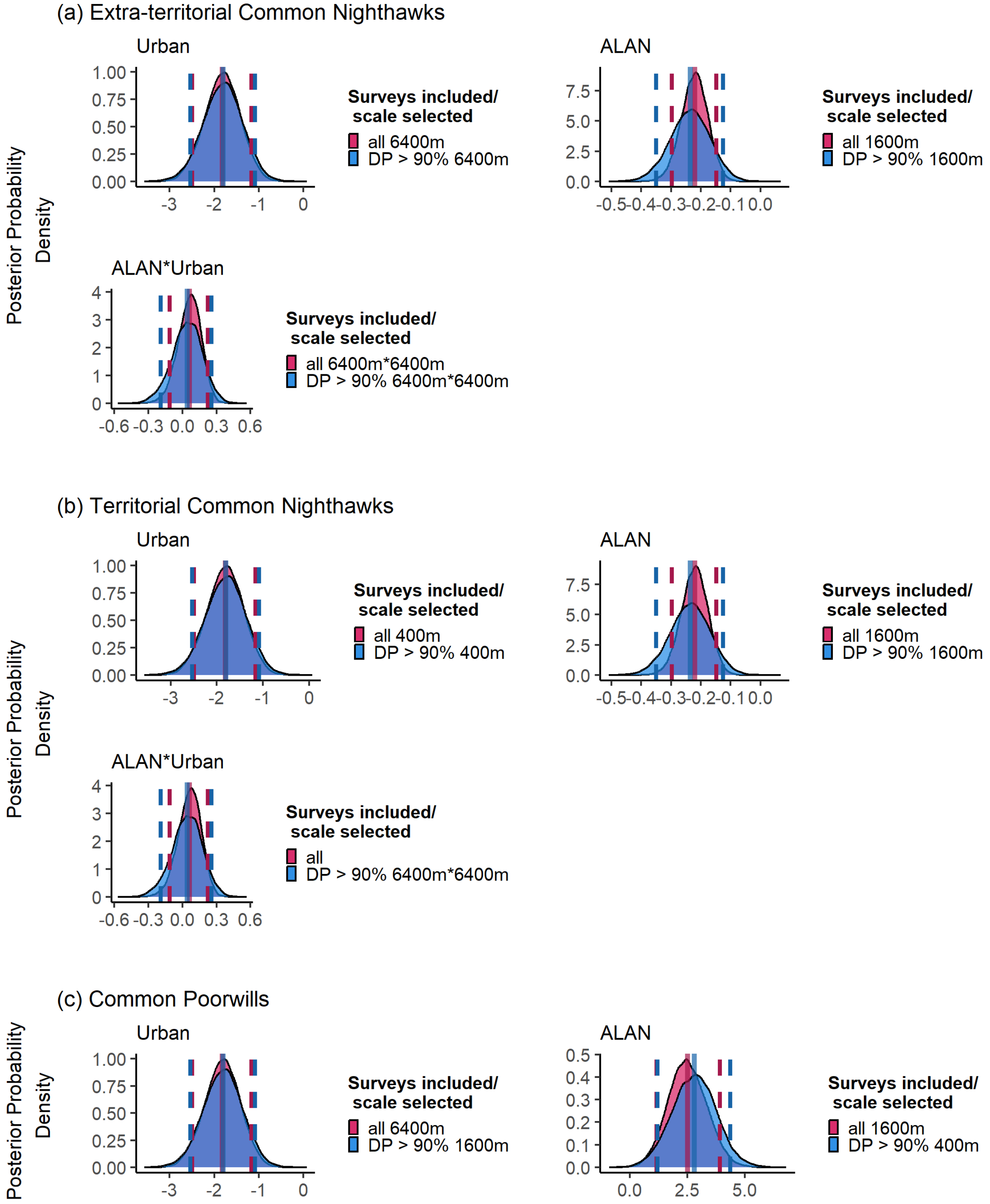

Figure S11 Examples of survey points with between 15% and 25% urban landcover within a 1600-meter buffer

In our model for extra-territorial Common Nighthawks, the effect of artificial light changed from positive to negative at when urban landcover at the 1600-m scale exceeded 18% (95% CI: 3%, 30%). These images are examples of 1600-m buffers around survey points where urban landcover is between 15% and 25%. These images from Esri World Imagery were generated in R using the *leaflet* package and the *Esri World Imagery* basemap.

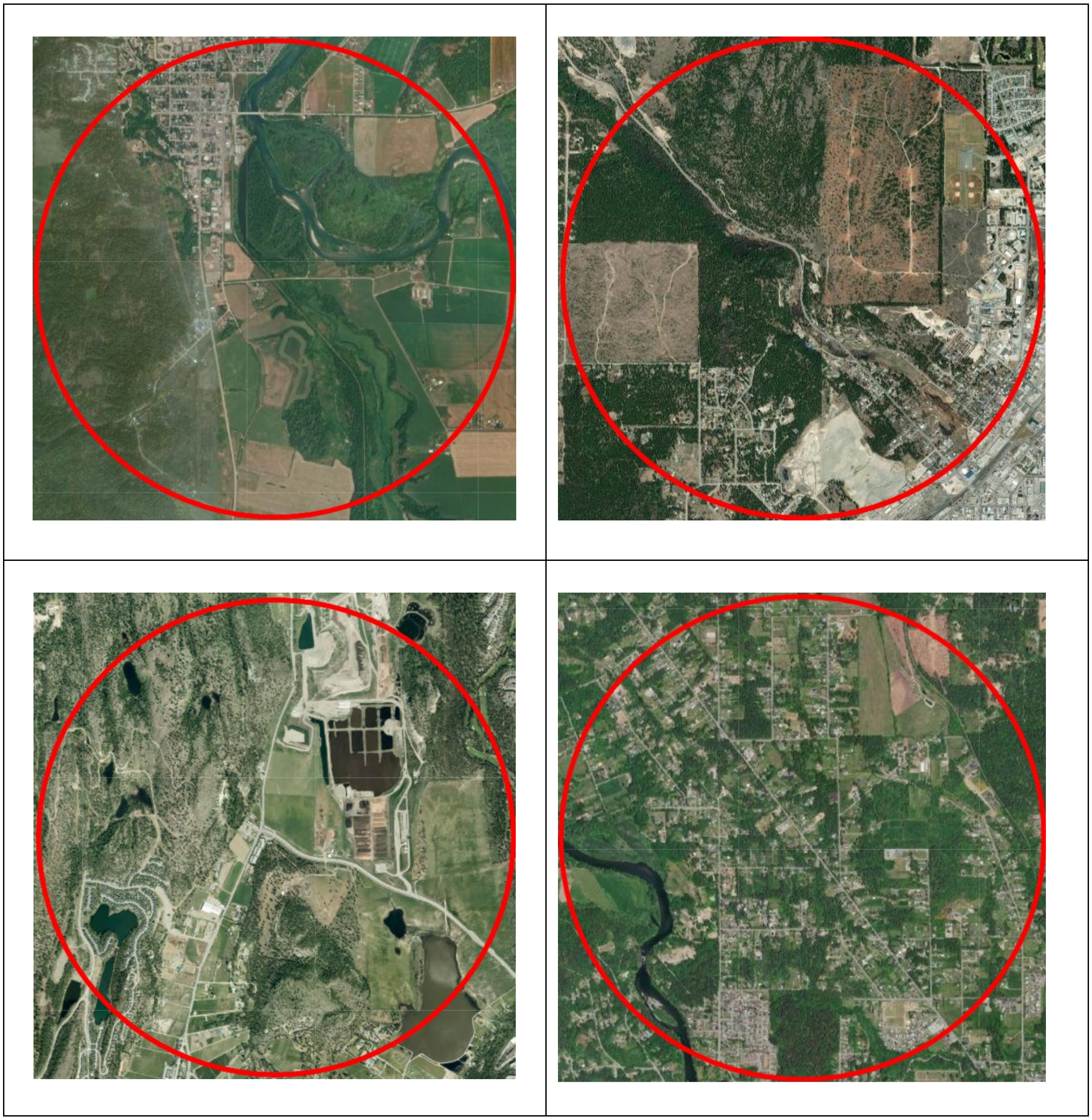

Supplement: Supplementary file 1 — Supplementary file1 (PDF 3344 kb) [file 10980_2024_1875_MOESM1_ESM.pdf]
